# The FNIP co-chaperones decelerate the Hsp90 chaperone cycle and enhance drug binding

**DOI:** 10.1038/ncomms12037

**Published:** 2016-06-29

**Authors:** Mark R. Woodford, Diana M. Dunn, Adam R. Blanden, Dante Capriotti, David Loiselle, Chrisostomos Prodromou, Barry Panaretou, Philip F. Hughes, Aaron Smith, Wendi Ackerman, Timothy A. Haystead, Stewart N. Loh, Dimitra Bourboulia, Laura S. Schmidt, W. Marston Linehan, Gennady Bratslavsky, Mehdi Mollapour

**Affiliations:** 1 Department of Urology, SUNY Upstate Medical University, 750 East Adams Street, Syracuse, New York 13210, USA; 2 Cancer Research Institute, SUNY Upstate Medical University, 750 East Adams Street, Syracuse, New York 13210, USA; 3 Department of Biochemistry and Molecular Biology, SUNY Upstate Medical University, 750 East Adams Street, Syracuse, New York 13210, USA; 4 Department of Pharmacology and Cancer Biology, Duke University Medical Center, Durham, North Carolina 27710, USA; 5 Genome Damage and Stability Centre, University of Sussex, Brighton BN1 9RQ, UK; 6 Institute of Pharmaceutical Science, King's College London, London SE1 9NH, UK; 7 Health Sciences Library, SUNY Upstate Medical University, 750 East Adams Street, Syracuse, New York 13210, USA; 8 Basic Science Program, Leidos Biomedical Research, Inc., Frederick National Laboratory for Cancer Research, Frederick, Maryland 21702, USA; 9 Urologic Oncology Branch, Center for Cancer Research, National Cancer Institute, 9000 Rockville Pike, Bethesda, Maryland 20892, USA

## Abstract

Heat shock protein-90 (Hsp90) is an essential molecular chaperone in eukaryotes involved in maintaining the stability and activity of numerous signalling proteins, also known as clients. Hsp90 ATPase activity is essential for its chaperone function and it is regulated by co-chaperones. Here we show that the tumour suppressor FLCN is an Hsp90 client protein and its binding partners FNIP1/FNIP2 function as co-chaperones. FNIPs decelerate the chaperone cycle, facilitating FLCN interaction with Hsp90, consequently ensuring FLCN stability. FNIPs compete with the activating co-chaperone Aha1 for binding to Hsp90, thereby providing a reciprocal regulatory mechanism for chaperoning of client proteins. Lastly, downregulation of FNIPs desensitizes cancer cells to Hsp90 inhibitors, whereas FNIPs overexpression in renal tumours compared with adjacent normal tissues correlates with enhanced binding of Hsp90 to its inhibitors. Our findings suggest that FNIPs expression can potentially serve as a predictive indicator of tumour response to Hsp90 inhibitors.

## Abstract (web summary)

Hsp90 is required for the folding, stability and activity of several drivers of oncogenesis. Here the authors show that Folliculin-interacting proteins (FNIP) 1 and 2, whose expression correlates with the cellular response to Hsp90 inhibitors, are co-chaperones of Hsp90 that function by inhibiting its ATPase activity.

## Graphical abstract

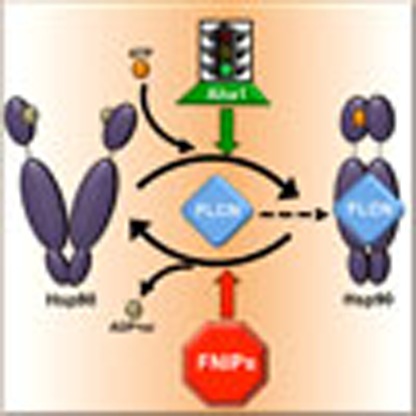

The molecular chaperone heat shock protein-90 (Hsp90) is responsible for folding, stability and activity of many proteins also known as ‘client proteins', including many responsible for tumour initiation, progression and metastasis^1^. This makes the chaperone Hsp90 an attractive target for cancer therapy^2^. Hsp90 has the ability to bind and hydrolyse ATP, which is essential for its chaperone function^3^. Small molecule inhibitors bind to the ATP-binding pocket of Hsp90 and inhibit its chaperone function. Consequently, this prevents Hsp90 interaction with client proteins, leading to their degradation by the proteasome. In contrast to other anticancer drugs, Hsp90 inhibitors simultaneously inhibit multiple drivers of oncogenesis.

Hsp90 chaperone cycle is tightly regulated by another group of proteins referred to as ‘co-chaperones'. Their stability does not depend on Hsp90 function but they interact with distinct Hsp90 conformational states, providing directionality to the Hsp90 cycle^4^. Furthermore, certain co-chaperones, such as HOP and Cdc37^p50^ inhibit the Hsp90 chaperone cycle, assisting in delivery of distinct sets of client proteins (steroid hormone receptors and kinases, respectively) to the Hsp90 chaperone machine. In contrast, the co-chaperone Aha1 facilitates energy-intensive conformational changes necessary to establish Hsp90 ATPase competence, markedly increasing the weak endogenous ATPase activity of Hsp90. Aha1 is thus considered to be a crucial component of active Hsp90 chaperone complexes^5^,^6^.

Here we show that the stability of the tumour suppressor folliculin (FLCN) depends on the chaperone function of Hsp90. Germline mutations and loss of function of FLCN causes Birt–Hogg–Dubé syndrome, a rare inherited cancer syndrome that predisposes affected individuals to develop kidney tumours, pulmonary cysts and benign skin tumours (fibrofolliculomas)^7^. FLCN interacts and forms a complex with folliculin-interacting proteins 1 and 2 (FNIP1 and FNIP2, also referred to as FNIPs)^8^,^9^,^10^. The function of FNIPs, however, remains elusive. Our results indicate that FNIPs act as co-chaperones of Hsp90. They inhibit its ATPase activity, ‘tailoring' Hsp90 to chaperone kinase and non-kinase clients. We have further shown that Aha1 co-chaperone can displace FNIPs and stimulate Hsp90 ATPase activity. Finally, FNIPs also enhance the binding of Hsp90 to its inhibitors such as ganetespib (GB); therefore, overexpression of FNIPs in specific tumours can be an indicator of their response to Hsp90 inhibitors.

## Results

### FLCN is a new client of Hsp90

**Figure 1:**
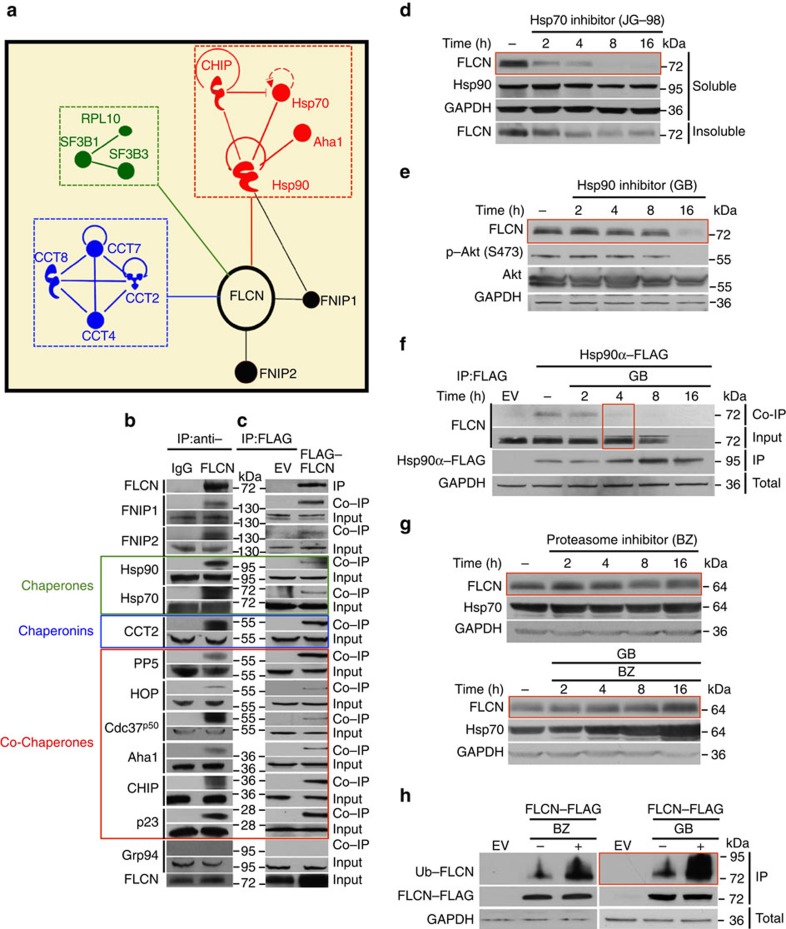
Folliculin is a new client of Hsp90. (**a**) FLAG–FLCN was expressed and isolated from HEK293 cells. Profile of interacting proteins determined by MALDI–time of flight. Red nodes represent chaperones and co-chaperones, blue nodes are chaperonins and green nodes are splicing factors and ribosomal proteins. (**b**) FLCN was isolated from HEK293 cell lysates using anti-FLCN or IgG (control) and immunoblotted with indicated antibodies to confirm protein interactions. (**c**) HEK293 cells were transiently transfected with FLAG–FLCN or empty vector control (EV), immunoprecipitated and immunoblotted with indicated antibodies to confirm interacting proteins. (**d**) HEK293 cells were treated with 10 μM of the Hsp70 inhibitor JG-98 at the indicated time points. FLCN protein stability in soluble and insoluble fraction was assessed by immunoblotting. (**e**) HEK293 cells were treated with 1 μM GB at the indicated time points. FLCN protein stability was assessed by immunoblotting. Akt and Phospho-S473-Akt were used as positive controls. (**f**) Hsp90α–FLAG was transiently expressed in HEK293 cells. Cells were treated with 1 μM GB for the indicated times. Hsp90α–FLAG was immunoprecipitated and co-IP of FLCN was examined by immunoblotting. (**g**) HEK293 cells were treated with 50 nM of the proteasome inhibitor bortezomib (BZ) for the indicated times. FLCN protein levels were evaluated at the indicated time points by immunoblotting (upper blots). HEK293 cells were also treated with 1 μM GB for 1 h before addition of 50 nM BZ. Immunoblotting was used to evaluate the FLCN level for the indicated time points (lower blots). (**h**) Empty vector (EV) or FLAG–FLCN was used to transiently transfect HEK293 cells for 24 h then treated for 4 h with either 50 nm BZ or 1 μM GB. FLAG–FLCN was immunoprecipitated and ubiquitination was examined by immunoblotting with a pan-anti-ubiquitin antibody.

To determine the binding partners of the tumour suppressor FLCN, we transiently expressed an amino-terminally FLAG-tagged FLCN (FLAG–FLCN) in human embryonic kidney 293 (HEK293) cells and identified its intracellular binding proteins by immunoprecipitating FLAG–FLCN with anti-FLAG M2 affinity gel and mass spectrometry (MS) analysis (Fig. 1a and Supplementary Table 1). We found molecular chaperones heat shock protein-70 (Hsp70) and Hsp90, and their regulators HOP, CHIP and Aha1, and CCT2, CCT4, CCT7 and CCT8, which are members of the chaperonin system TRiC (TCP-1 ring complex), (Fig. 1a). We validated our data by immunoprecipitating the endogenous FLCN (Fig. 1b) or the FLAG–FLCN (Fig. 1c) from HEK293 cells and showed its interaction with the molecular chaperone machineries Hsp70, Hsp90 and a subunit of the chaperonin TRiC, CCT2 (Fig. 1b,c). We also observed FLCN interaction with the Hsp70 and Hsp90 co-chaperones including HOP, CHIP, Cdc37^p50^, PP5, p23 and Aha1 (Fig. 1b,c). In general, molecular chaperones are involved in folding and stability of proteins. We first treated the HEK293 cells with the Hsp70 inhibitor JG-98 (ref. 11) and showed the degradation of FLCN after a 2h treatment in both soluble and insoluble protein fractions (Fig. 1d). These data suggest that inhibition of Hsp70 does not lead to an increase in misfolded FLCN but instead to its degradation. The molecular chaperone Hsp90 however is more selective towards its ‘client proteins' and is also involved in protecting them from degradation^12^. Therefore, we treated the HEK293 cells with different inhibitors of Hsp90 such as GB^13^ (Fig. 1e), SNX2112 (ref. 14) and PU-H71 (ref. 15) (Supplementary Fig. 1a,b), to evidence the degradation of FLCN. Previous works have shown that inhibition of Hsp90 generally leads to ubiquitination and degradation of its client proteins in the proteasome^16^. We investigated this possibility by first demonstrating that inhibition of Hsp90 causes its dissociation from FLCN (Fig. 1f). We further showed that HEK293 cells treated with 50 nM proteasome inhibitor bortezomib for 2 h before addition of GB blocked FLCN degradation (Fig. 1g). We did not obtain similar results when we treated the cells with the lysosomal inhibitor Bafilomycin A1 (Supplementary Fig. 1c), suggesting that the lysosome is not involved in degradation of FLCN. We next immunoprecipitated and salt-stripped (with 0.5 M NaCl) FLAG–FLCN from HEK293 cells treated with either 50 nM bortezomib or 1 μM GB for 4 h and showed its ubiquitination by western blotting (Fig. 1h). Taken together, our results suggest that FLCN is a client of Hsp70 and Hsp90. Inhibition of Hsp90 leads to ubiquitination and degradation of FLCN in the proteasome.

### FNIP1 and FNIP2 facilitate FLCN binding to Hsp90 chaperone

**Figure 2:**
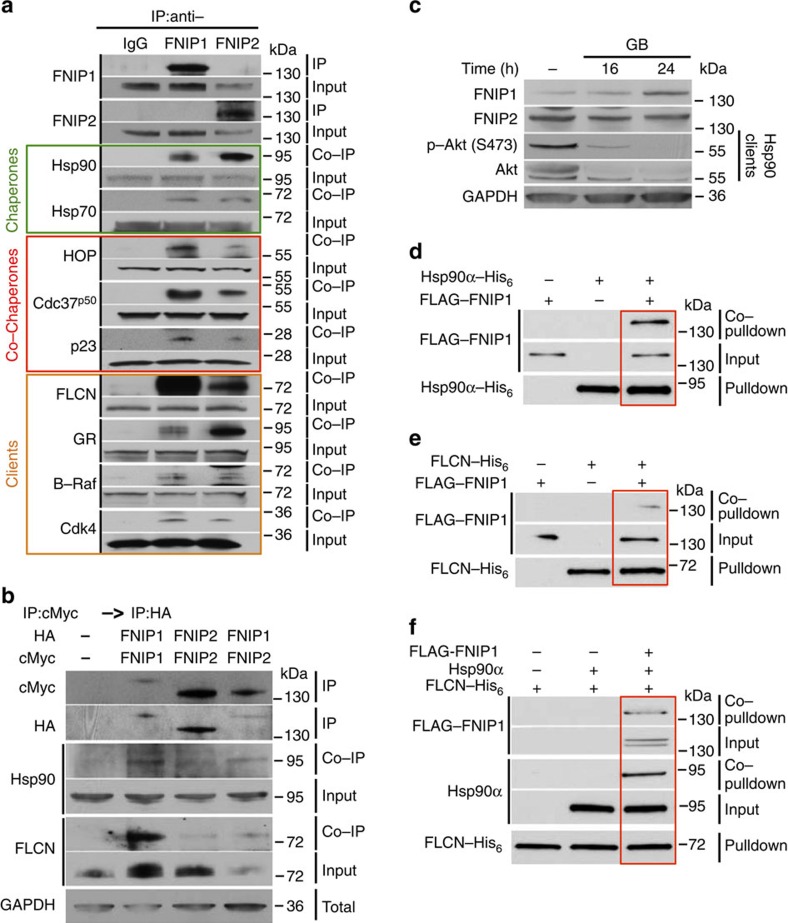
FNIPs facilitate FLCN binding to the Hsp90 chaperone. (**a**) FNIP1 and FNIP2 were isolated from HEK293 cell lysates using anti-FNIP1, anti-FNIP2 or IgG (control) and immunoblotted with indicated antibodies to confirm protein interaction. (**b**) HA–FNIP1 and HA–FNIP2 were transiently co-expressed with either cMyc–FNIP1 or cMyc–FNIP2 in HEK293 cells. cMyc–FNIP1 and cMyc–FNIP2 were first isolated followed by IP of HA–FNIP1 and HA–FNIP2 from the same samples. The quality of HA–FNIP1:cMyc–FNIP1 and HA–FNIP2:cMyc–FNIP2 homodimers and HA–FNIP1:cMyc–FNIP2 heterodimer were assessed by immunoblotting. Co-IP of Hsp90 and FLCN was also assessed by western blotting. (**c**) HEK293 cells were treated with 1 μM GB for the indicated times, and FNIP1 and FNIP2 protein levels were determined by immunoblotting. Akt and Phospho-S473-Akt were used as positive controls. (**d**) FLAG–FNIP1 interacts with Hsp90α–His_6_ *in vitro*. Bacterially expressed and purified Hsp90α–His_6_ was bound to Ni-NTA agarose and then incubated with 10 ng pure FLAG–FNIP1. Hsp90α–His_6_ pulldown and FLAG–FNIP1 co-pulldown were assessed by immunoblotting. (**e**) FLAG–FNIP1 interacts with FLCN–His_6_ *in vitro*. Bacterially expressed and purified FLCN–His_6_ was bound to Ni-NTA agarose and then incubated with 10 ng pure FLAG–FNIP1. FLCN–His_6_ pulldown and FLAG–FNIP1 co-pulldown were assessed by immunoblotting. (**f**) FLCN–His_6_, FLAG–FNIP1 and Hsp90α tri-complex *in vitro.* Bacterially expressed and purified FLCN–His_6_ was bound to Ni-NTA agarose and followed by incubation with 10 ng FLAG–FNIP1 and then 10 ng untagged Hsp90α *in vitro*. FLCN–His_6_ pulldown, and FLAG–FNIP1 and Hsp90α co-pulldown were examined by immunoblotting.

FNIP1 and FNIP2 are homologous binding partners of FLCN^8^,^10^; however, their molecular function remains elusive. In addition, FNIP1 was shown to interact with Hsp90, but the significance of this observation was not researched further^8^. We first confirmed these data by immunoprecipitating the endogenous FNIP1 and FNIP2 from HEK293 cells and detecting Hsp90 (Fig. 2a). We also observed FNIPs interaction with Hsp70 and co-chaperones p23, HOP and Cdc37^p50^ (Fig. 2a). In addition, we co-immunoprecipitated Hsp90 clients such as glucocorticoid receptor (GR), B-Raf, Cdk4 and FLCN (Fig. 2a). We confirmed our data by transiently expressing and immunoprecipitating HA–FNIP1 and HA–FNIP2 from HEK293 cells and then probing for the chaperones, co-chaperone and client proteins (Supplementary Fig. 2a). Previous work has shown that FNIPs are homo- and heterodimer proteins^10^. We isolated FNIP1 and FNIP2 homo- and heterodimers by transiently co-expressing cMyc–FNIP1/HA–FNIP1, cMyc–FNIP2/HA–FNIP2 and HA–FNIP1/cMyc–FNIP2 in HEK293 cells. We first immunoprecipitated cMyc-tagged proteins using anti-cMyc agarose affinity gel. We then competed the cMyc proteins from the affinity gel with cMyc peptides. The eluted proteins were dialysed and subjected to a second round of immunoprecipitation (IP) using anti-HA agarose affinity gel. Immunoprecipitated proteins have shown that FNIP1 homodimer has a stronger affinity for binding to Hsp90 and FLCN than FNIP2 (Fig. 2b). We next asked whether FNIP1 or FNIP2 are clients or co-chaperones of Hsp90. Treating HEK293 cells with the Hsp90 inhibitor GB did not affect FNIP protein stability (Fig. 2c). These data suggest that FNIP1 and FNIP2 are co-chaperones of Hsp90. We obtained further evidence by demonstrating a direct interaction between FNIP1 and Hsp90. We were able to bacterially express and purify a small amount of FLAG–FNIP1. Our purified FLAG–FNIP1 can directly interact with bacterially expressed and purified Hsp90α–His_6_ (Fig. 2d) and FLCN–His_6_ (Fig. 2e). Surprisingly, Hsp90α and FLCN did not directly interact with each other *in vitro*; however, pre-incubation of Hsp90α with FNIP1 facilitated the Hsp90–FNIP1–FLCN complex formation (Fig. 2f). It is noteworthy that we were unable to express and purify an adequate amount of FNIP2 protein from bacteria, yeast or baculovirus expression systems for biophysical analysis.

**Figure 3:**
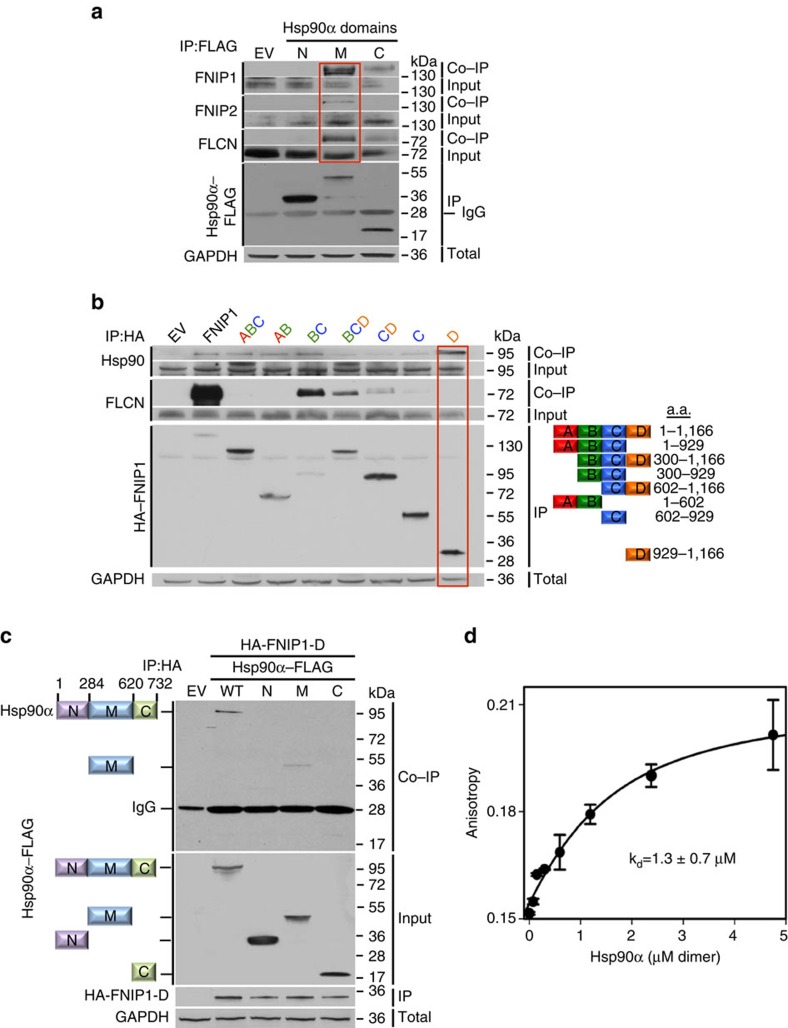
FNIPs directly bind to Hsp90 M- and C-domains. (**a**) FLAG-tagged Hsp90α-N-, M-, and C-domains were transiently expressed and immunoprecipitated from HEK293 cells. Co-IP of endogenous FLCN and FNIPs were assessed by immunoblotting. Empty vector (EV) was used as a control. (**b**) HA-tagged FNIP1 domains were transiently expressed and isolated from HEK293 cells. Co-IP of endogenous FLCN and Hsp90 were assessed by immunoblotting. Empty vector (EV) was used as a control. (**c**) FLAG-tagged Hsp90α domains were transiently co-expressed with HA-tagged FNIP1-D in HEK293 cells. HA–FNIP1-D was immunoprecipitated and Hsp90α–FLAG co-IP was evaluated by immunoblotting. (**d**) Bacterially expressed and purified FNIP1-D and Hsp90α binding affinity measured by fluorescence anisotropy. The error bars represent mean ±s.d. of three independent experiments.

Hsp90 consists of N (amino), M (middle) and C (carboxy) domains. To determine FNIP and FLCN interaction with these Hsp90 domains, we transiently expressed each domain with FLAG-tag in HEK293 cells. Following IP with anti-FLAG M2 affinity gel, we observed FNIP1 interaction with the Hsp90α M- and C-domains, whereas FNIP2 interacted with only the M-domain of Hsp90α (Fig. 3a). Association of FLCN with the M- and C-domains of Hsp90α was also observed (Fig. 3a).

We next determined the region in FNIP1 that interacts with Hsp90. FNIP1 does not have a known functional domain; however, based on previous studies^8^,^10^, we constructed and transiently expressed four regions of HA–FNIP1 designated A–D in HEK293 cells (Fig. 3b). Consistent with the previous work^8^,^10^, truncation of FNIP1 at the C-terminus (deletion of fragment D) abrogated its interaction with FLCN (Fig. 3b). However, the concomitant deletion of 300 amino acids (fragment A) from the N-terminus of FNIP1 restored its binding to FLCN (Fig. 3b). This result is not in agreement with the previous study^8^ and underlies the complexity in FNIP1 binding to FLCN. Our data also indicated that the C-domain of FNIP1 (amino acid 929–1166 or fragment D) preferentially interacts with Hsp90 (Fig. 3b). We confirmed these data by carrying out a reciprocal experiment and co-expressing FNIP1-D–HA and Hsp90α–FLAG full-length (wild type) and its different domains in HEK293 cells. We immunoprecipitated FNIP1-D–HA and observed co-IP of Hsp90α full length and its M-domain (Fig. 3c). We next bacterially expressed and purified fragment D (FNIP1-D–His_6_), (Supplementary Fig. 2b,c) and examined its affinity for binding to Hsp90α *in vitro* by fluorescently labelling FNIP1-D–His_6_ with Texas Red Maleimide, and measured the *K*_d_ by fluorescence anisotropy (Fig. 2j). Our bacterially expressed and purified HSP90α had ATPase activity. The titration fit to a single-site binding equation with a *K*_d_ of 1.3±0.7 μM (Fig. 3d). This *K*_d_ was unaffected by the presence, absence or identity of adenosine nucleotide bound to the protein (Supplementary Fig. 2d). Taken together, our findings suggest that FNIPs behave as co-chaperones of Hsp90 and they are involved in loading of FLCN to Hsp90.

### FNIPs regulate Hsp90 activity and chaperoning of the clients

**Figure 4:**
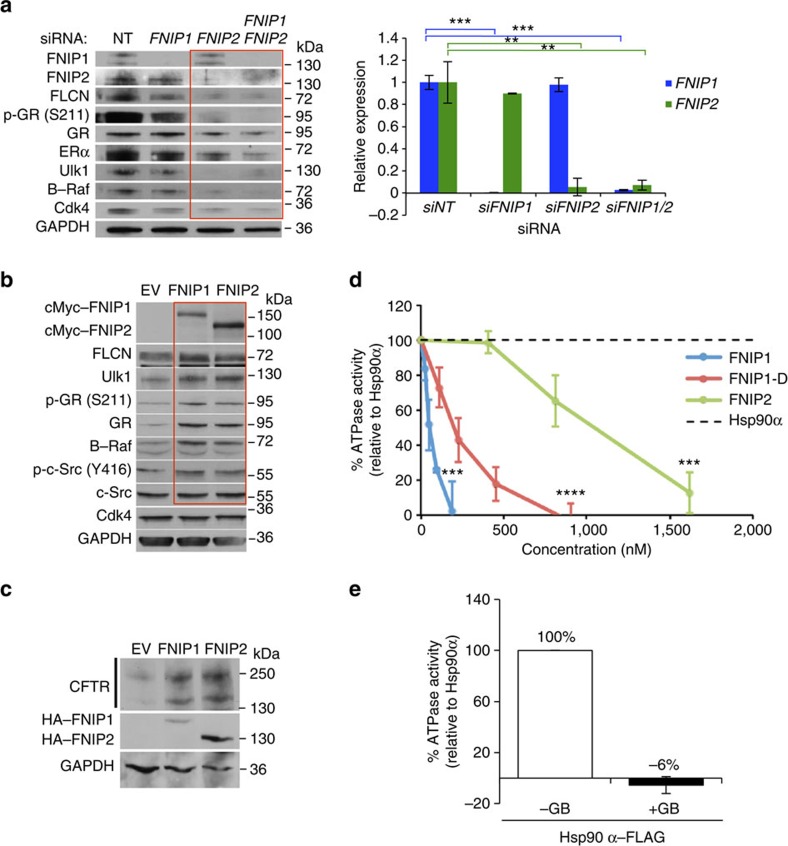
FNIPs co-chaperones inhibit Hsp90 chaperone cycle and facilitate chaperoning of the clients. (**a**) Effect of siRNA knockdown of *FNIP1* and *FNIP2* on Hsp90 clients. Stability and activity of the indicated clients were assessed by immunoblotting. Densitometry of the western blotting for FNIP1 and FNIP2 is represented as mean±s.d. A Student's *t*-test was performed to assess statistical significance (**P<0.005 and ***P<0.0005). (**b**) Transient overexpression of cMyc-tagged FNIP1 or FNIP2 in HEK293 cells and their impact on levels of Hsp90 clients was assessed by immunoblotting. Empty vector (EV) was used as a control. (**c**) HEK293 cells were co-transfected with CFTR and indicated HA–FNIP1 and HA–FNIP2 constructs. After 24 h, CFTR and FNIPs–HA were detected by immunoblotting; GAPDH was used as loading control. Empty vector (EV) was used as a control. (**d**) *In vitro* ATPase activity of Hsp90α–FLAG isolated from PC3 cells. Inhibitory effects of purified HA–FNIP1, FNIP1-D–HA or HA–FNIP2 on ATPase activity of Hsp90α–FLAG. All the data represent mean±s.d. A Student's *t*-test was performed to assess statistical significance (***P<0.0005 and ****P<0.0001). (**e**) ATPase activity of Hsp90α–FLAG from **d** was inhibited by addition of 10 μM GB. All the data represent mean±s.d. A Student's *t*-test was performed to assess statistical significance (****P<0.0001).

To gain further insight into FNIP1 and FNIP2 function as co-chaperones of Hsp90, we used small interfering RNA (siRNA) to silence either *FNIP1* or *FNIP2* (Supplementary Table 2), or both in HEK293 cells, and monitored the stability and activity of the Hsp90 kinase clients such as B-Raf, Ulk1 and Cdk4, and non-kinase clients, for example, GR, ER and FLCN (Fig. 4a and Supplementary Fig. 3a). Silencing either *FNIP1* or *FNIP2* caused a modest decrease in client protein levels. This data suggests that the absence of either *FNIP1* or *FNIP2* allows for compensation by the other FNIP isoform. Silencing of both *FNIP1* and *FNIP2* significantly decreased the stability of the selected kinase and non-kinase client proteins (Fig. 4a). We next transiently overexpressed cMyc–FNIP1 or cMyc–FNIP2 in HEK293 cells and examined the stability and the activity of the Hsp90 clients. Overexpression of FNIPs caused an increase in GR, Ulk1 and FLCN protein levels and hyperphosphorylation of B-Raf and pY416-c-Src (Fig. 4b and Supplementary Fig. 3b).

Cystic fibrosis transmembrane conductance regulator (CFTR) is also an Hsp90 client 17,18 and relies on a ‘slow' Hsp90 chaperone cycle for proper folding. To examine the effect of FNIPs on Hsp90 chaperone function, we assessed their impact on steady-state expression of CFTR protein in mammalian cells. HEK293 cells were transiently co-transfected with CFTR, and HA–FNIP1 and HA–FNIP2. Empty plasmid pcDNA3 (empty vector, EV) was used as a negative control. Western blot analysis of these samples using anti-CFTR antibody detected a doublet (Fig. 4c), with the upper band representing the mature Golgi-processed glycoform of CFTR found at the cell surface and the lower band an immature core-glycosylated protein (Fig. 4c). Overexpression of the FNIPs produced a significant increase in CFTR protein (Fig. 4c). These data suggested a reduction in Hsp90 chaperone activity and, therefore, an increase in CFTR expression.

Hsp90 chaperone function is coupled to its ATPase activity^3^. We therefore examined the impact of FNIPs on Hsp90 ATPase activity. We transiently expressed Hsp90α–HA in the prostate cancer PC3 cell line and HA–FNIP1, HA–FNIP1-D (amino acids 929–1166) and HA–FNIP2 in HEK293 cells. Proteins were immunoprecipitated, salt stripped and competed off the HA affinity beads with the relevant peptides (Supplementary Fig. 4a). The quality of the isolated proteins was examined by Coomassie staining of the SDS–polyacrylamide gel electrophoresis (SDS–PAGE). These purified proteins were quantified and used in the molar ratio indicated in Supplementary Fig. 4b of Hsp90α:FNIP in the PiPer Phosphate Assay Kit (Thermo Fisher Scientific), in the presence of ATP as substrate as previously described (see Methods)^19^. We measured the ATPase activity of isolated Hsp90α *in vitro* as previously described^19^ (Fig. 4d and Supplementary Fig. 4c). GB (10 μM) inhibited ATPase activity (Fig. 4e). Addition of FNIP1, FNIP1-D and FNIP2 also significantly inhibited the ATPase activity of Hsp90α (Fig. 4d and Supplementary Fig. 4c). Percentage ATPase activity was based on mmol *P*_i_ per mol min^−1^ for Hsp90α alone and in the presence of FNIPs titrated until inhibition was achieved (Hsp90α=291.5 nM, FNIP1=189.8 nM, FNIP1-D=899.3 nM and FNIP2=1.6 μM) (Supplementary Fig. 4d). These data suggest that FNIPs are potent inhibitors of the Hsp90 chaperone cycle.

**Figure 5:**
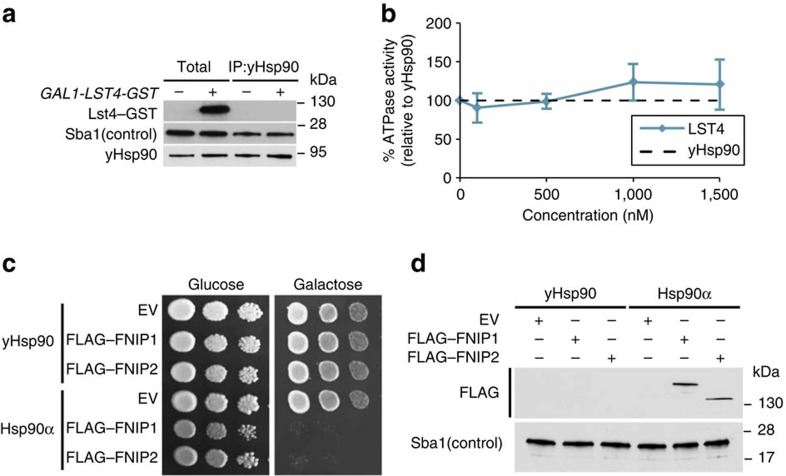
Lst4 is not an orthologue of FNIPs in yeast. (**a**) BY4741 yeast strain expressing *GAL1-LST4-GST* was grown on either YPED (glucose) or YPGal (Galactose) media at 30 °C for 8 h. Endogenous yHsp90 immunoprecipitation (IP) and co-IP Lst4-GST was detected by immunoblotting. (**b**) *In vitro* ATPase activity of the yHsp90–His_6_ with indicated amounts Lst4-GST. All the data represent mean±s.d. (**c**) PP30 strain expressing either yHsp90 or Hsp90α as the sole copies of Hsp90 were transformed by *GAL1–FLAG–FNIP1* or *GAL1–FLAG–FNIP2.* Cells were spotted at 1:10 dilution series on YPED or YPGal media. Plates were incubated at 30 °C for 2 days. (**d**) Yeast strains in **i** were grown on YPGal liquid media for 12 h and the expression of FLAG–FNIP1 and FLAG–FNIP2 were examined by immunoblotting.

Hsp90 is an evolutionarily conserved chaperone and based on structural homology detection methods Lst4 is a potential orthologue of FNIPs in *Saccharomyces cerevisiae*^20^. However, we were unable to detect Lst4–GST interaction with yeast Hsp90 (yHsp90, also known as Hsp82) (Fig. 5a). In addition, unlike FNIPs, Lst4–GST was unable to inhibit ATPase activity of yHsp90 *in vitro* (Fig. 5b and Supplementary Fig. 4e,f). We next decided to express *FLAG–FNIP1* and *FLAG–FNIP2* galactose-inducible promoter of *GAL1* in yeast containing either human or yeast Hsp90 as the sole functional Hsp90. Overexpression of FNIPs in yeast containing human Hsp90α caused lethality (Fig. 5c). However, overexpression of FNIPs in yeast with only yHsp90 did not cause any growth defects. We examined the expression of the FNIPs in these cells by western blot analysis and were only able to detect FLAG–FNIP1 and FLAG–FNIP2 in yeast expressing Hsp90α (Fig. 5d). These data suggest that expression of FNIPs in yeast cause lethality, because they inhibit the essential chaperone function of Hsp90α. Taken together, our results suggest that (i) Lst4 is not an orthologue of FNIPs in yeast and (ii) FNIPs probably arose in higher eukaryotes as co-chaperones to inhibit or slow the complex chaperone cycle of Hsp90 in cellular milieu.

### FNIPs compete with the Aha1 co-chaperone for binding to Hsp90

**Figure 6:**
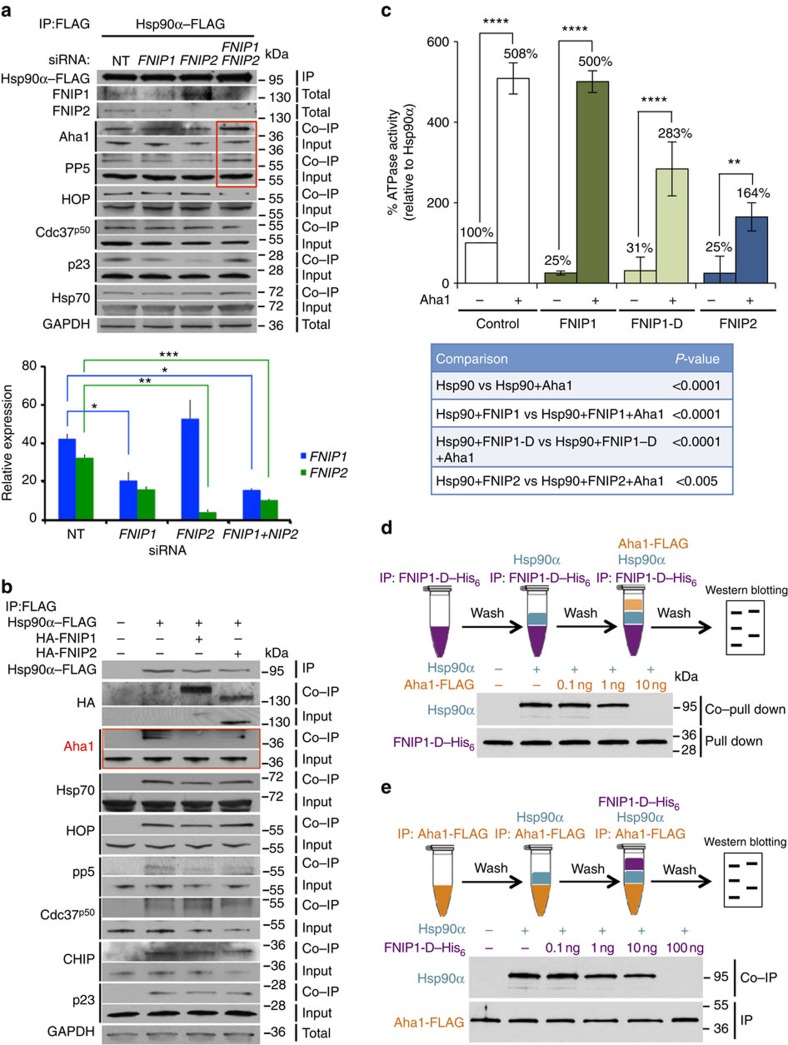
FNIPs compete with the Aha1 co-chaperone for binding to Hsp90. (**a**) Hsp90α–FLAG was transiently expressed in HEK293 cells for 24 h followed by siRNA knockdown of *FNIP1* and/or *FNIP2*. Hsp90α–FLAG was immunoprecipitated (IP) and co-IP of the co-chaperones was assessed by immunoblotting. Densitometry of the western blotting for FNIPs is represented as mean±s.d. A Student's *t*-test was performed to assess statistical significance (*P<0.05, **P<0.005 and ***P<0.0005). (**b**) HEK293 cells were transiently co-transfected with Hsp90α–FLAG and HA–FNIP1 or HA–FNIP2. Hsp90α–FLAG was isolated and co-IP of co-chaperones examined by immunoblotting. (**c**) HA–FNIP1, HA–FNIP1-D and HA–FNIP2 inhibited Hsp90α–HA ATPase activity after 30 min. Addition of 1.3 μM Aha1–FLAG stimulated the ATPase activity. All the data represent mean±s.d. A Student's *t*-test was performed to assess statistical significance (**P<0.005 and ****P<0.0001). (**d**) FNIP1 and Aha1 compete for binding to Hsp90α. FNIP1-D–His_6_ was attached to Ni-NTA agarose and then incubated with Hsp90α. Ni-NTA agarose was then washed and incubated with the indicated amounts of Aha1–FLAG. (**e**) Aha1–FLAG attached to anti-FLAG M2 affinity gel was incubated with Hsp90α initially and then washed and incubated with indicated amounts of the FNIP1-D–His_6_.

To determine the effects of FNIPs on Hsp90 interaction with co-chaperones, we used *FNIP1*- and *FNIP2*-specific siRNA to silence expression of these genes in HEK293 cells transiently expressing Hsp90α–FLAG. We next immunoprecipitated Hsp90α–FLAG from these cells and examined its interaction with selected co-chaperones. Our data show that siRNA-mediated silencing of both *FNIP1* and *FNIP2* increased Hsp90 interaction with the co-chaperones Aha1 and PP5 (Fig. 6a). We next transiently overexpressed HA–FNIP1 and HA–FNIP2 in HEK293 cells that also express Hsp90α–FLAG. The overexpression of FNIPs compared with the endogenous FNIP1 and FNIP2 were examined by immunoblotting (Supplementary Fig. 5a). Following IP of Hsp90α–FLAG, we observed a marked reduction in its interaction with the co-chaperones Aha1 and PP5 (Fig. 6b). Aha1 is the activator of the Hsp90 ATPase activity^5^,^6^. We next queried whether Aha1 has the ability to stimulate the ATPase activity of Hsp90 that has already bound to FNIP1, FNIP1-D or FNIP2. In agreement with our earlier data, FNIPs inhibited the ATPase activity of Hsp90α and, conversely, addition of 1.3 μM Aha1 to these reactions stimulated Hsp90 ATPase activity (Fig. 6c and Supplementary Fig. 5b–f). Taken together, these data suggest that Aha1 has the ability to displace FNIPs from Hsp90. We obtained additional evidence for this possibility by using the bacterially expressed and purified Hsp90α, Aha1–FLAG and FNIP1-D fragment. First, we bound FNIP1-D–His_6_ to Ni-NTA agarose and incubated with 100 ng recombinant Hsp90α. After washing the agarose with buffer, we added different amounts of Aha1–FLAG. Our data show that addition of 10 ng recombinant Aha1–FLAG completely dissociated Hsp90α from FNIP1-D–His_6_ (Fig. 6d). We next carried out a similar experiment by first immobilizing Aha1–FLAG onto anti-FLAG M2 affinity gel. We then added 100 ng recombinant Hsp90α, to form an Aha1–Hsp90α complex. After washing the agarose beads with buffer, we added different amounts of FNIP1-D–His_6_. We observed that addition of 100 ng FNIP1-D–His_6_ disrupted the Aha1–Hsp90α complex (Fig. 6e). We repeated the experiments in Fig. 6d,e, but using bovine serum albumin as a negative control (Supplementary Fig. 5g–i). Our data confirm that Aha1 and FNIP1 compete for binding to Hsp90, either accelerating or decelerating its chaperone cycle. In addition, Aha1 appears to have higher affinity than FNIP1 towards Hsp90, as 10 ng of Aha1 was sufficient to displace FNIP1-D from Hsp90 (Fig. 6d).

### FNIPs overexpression enhances Hsp90 binding to ATP and drugs

**Figure 7:**
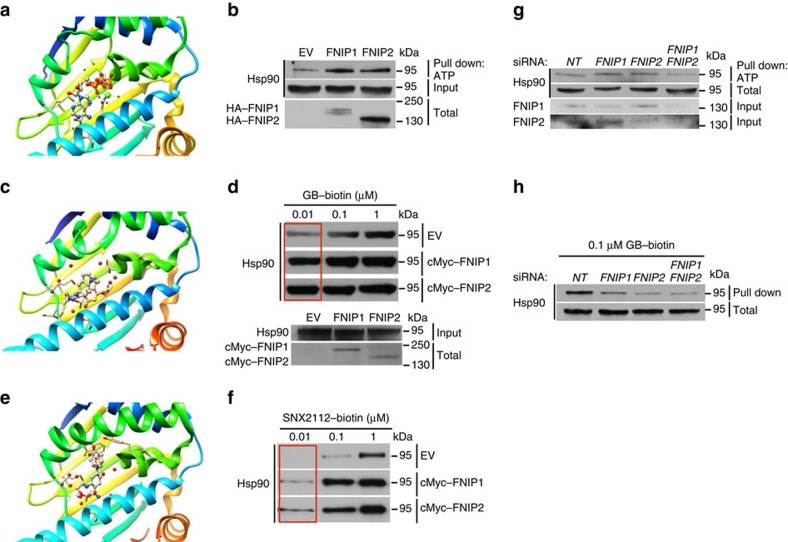
Overexpression of FNIPs enhance Hsp90 binding to drugs. (**a**) Structure of human Hsp90α N-domain bound to ATP (PDB: 3T0Z). (**b**) HA–FNIP1, HA–FNIP2 or empty vector (EV) were transiently overexpressed in HEK293 cells. Lysates were incubated with ATP agarose. Hsp90 binding to ATP agarose was examined by immunoblotting. (**c**) Structure of human Hsp90α N-domain bound to GB (PDB: 3TUH). (**d**) cMyc–FNIP1, cMyc–FNIP2 or empty vector (EV) were transiently overexpressed in HEK293 cells. Lysates were incubated with indicated amounts of biotinylated GB followed by streptavidin agarose beads. Hsp90 was detected by immunoblotting. (**e**) Structure of human Hsp90α bound to related SNX2112 compound, tetrahydro-4H-carbazol-4-one (PDB: 3D0B). (**f**) Lysates from **d** were incubated with indicated amounts of biotinylated SNX2112 followed by streptavidin agarose beads. Hsp90 was detected by immunoblotting. (**g**) *FNIP1* and *FNIP2* were silenced by siRNA in HEK293 cells. Lysates were incubated with ATP agarose. Hsp90 binding to ATP agarose was examined by immunoblotting. NT represents non-targeting siRNA control pools. (**h**) Lysates from **g** were incubated with indicated amounts of biotinylated GB followed by streptavidin agarose beads. Hsp90 was detected by immunoblotting.

ATP binding and hydrolysis by the N-terminus of Hsp90 is essential for its chaperone function (Fig. 7a) 3. We examined the impact of FNIPs on Hsp90 binding to ATP or drugs by first overexpressing HA–FNIP1 and HA–FNIP2 in HEK293 cells. Overexpression of FNIPs caused an increase in Hsp90 binding to ATP agarose (Fig. 7b). Furthermore, overexpression of cMyc–FNIP1 and cMyc–FNIP2 in HEK293 cells significantly increased the Hsp90 binding to 0.01 μM biotinylated-GB (Fig. 7c,d) and 0.01 μM biotinylated-SNX2112 (ref. 14 and Fig. 7e,f) compared with control (EV). We next examined the effects of *FNIP1* and *FNIP2* knockdown on Hsp90 binding to ATP and drug. siRNA knockdown of *FNIP1*, *FNIP2* or both in HEK293 cells did not have an impact on Hsp90 binding to ATP (Fig. 7g) but significantly decreased Hsp90 binding to GB (Fig. 7h).

**Figure 8:**
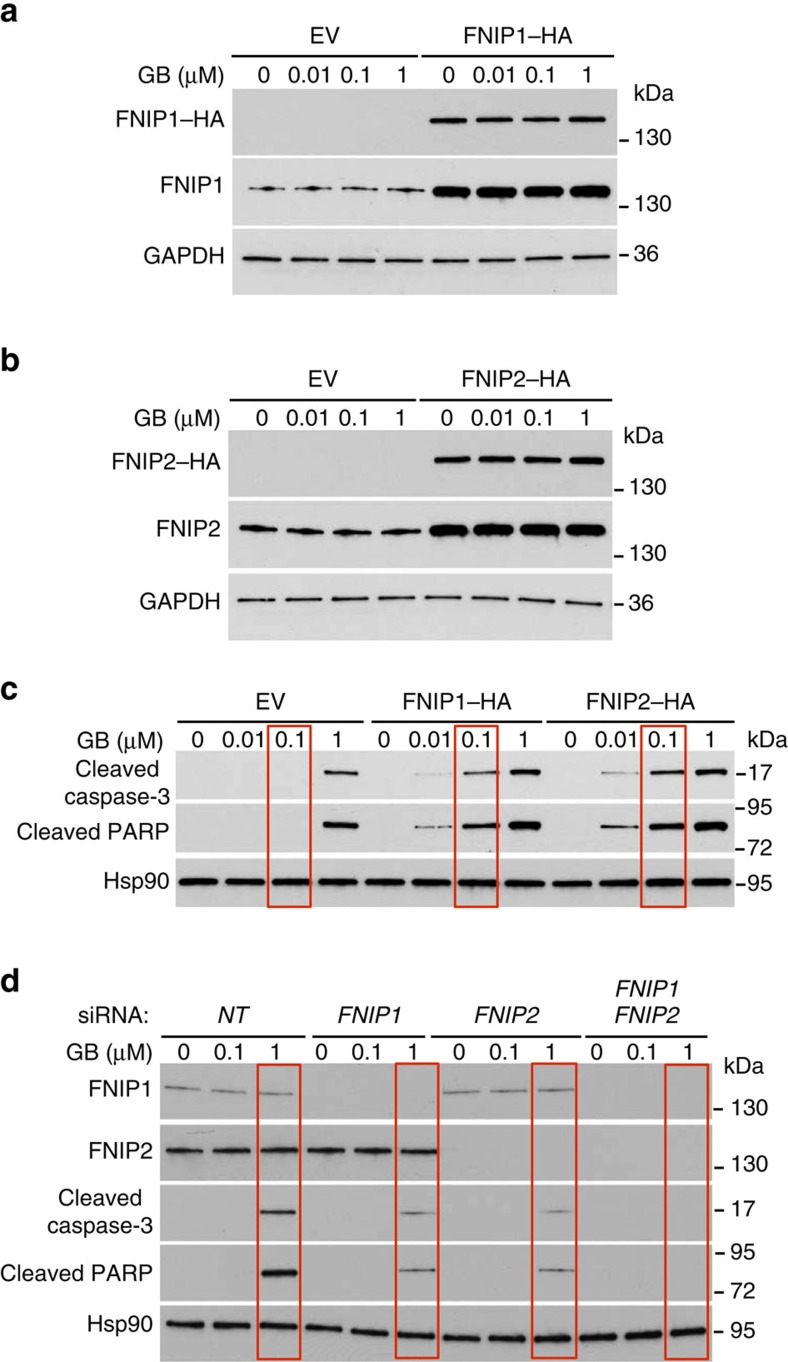
Overexpression of FNIPs sensitize HEK293 cells to Hsp90 inhibitor. (**a**) HEK293 cells were transiently transfected with HA–FNIP1 or empty vector (EV), (**b**) or HA–FNIP2 or empty vector (EV), and treated with indicated amounts of GB. (**c**) Induction of apoptotic markers in lysates from **a** and **b** were shown by immunoblotting using anti-cleaved caspase-3 and cleaved PARP antibodies. (**d**) *FNIP1*, *FNIP2* or both were silenced by siRNA in HEK293 cells and then treated with indicated amounts of GB. Apoptotic markers were examined by immunoblotting using anti-cleaved caspase-3 and cleaved PARP antibodies.

We next confirmed that overexpression of either *FNIP1–HA* or *FNIP2–HA* (Fig. 8a,b) sensitized the HEK293 cells to GB, as evidenced by induction of the pro-apoptotic markers cleaved caspase-3 and cleaved poly-ADP-ribose polymerase (PARP) (Fig. 8c). Conversely, siRNA knockdown of *FNIP1* or *FNIP2* in HEK293 cells significantly reduced apoptosis with 1 μM GB and knockdown of both *FNIPs* completely abolished the induction of apoptotic markers (Fig. 8d). Taken together, these data suggest that expression of FNIPs correlates to Hsp90 binding to drugs and also sensitivity of cells to Hsp90 inhibitors.

### FNIPs overexpression sensitizes cancer cells to Hsp90 drugs

**Figure 9:**
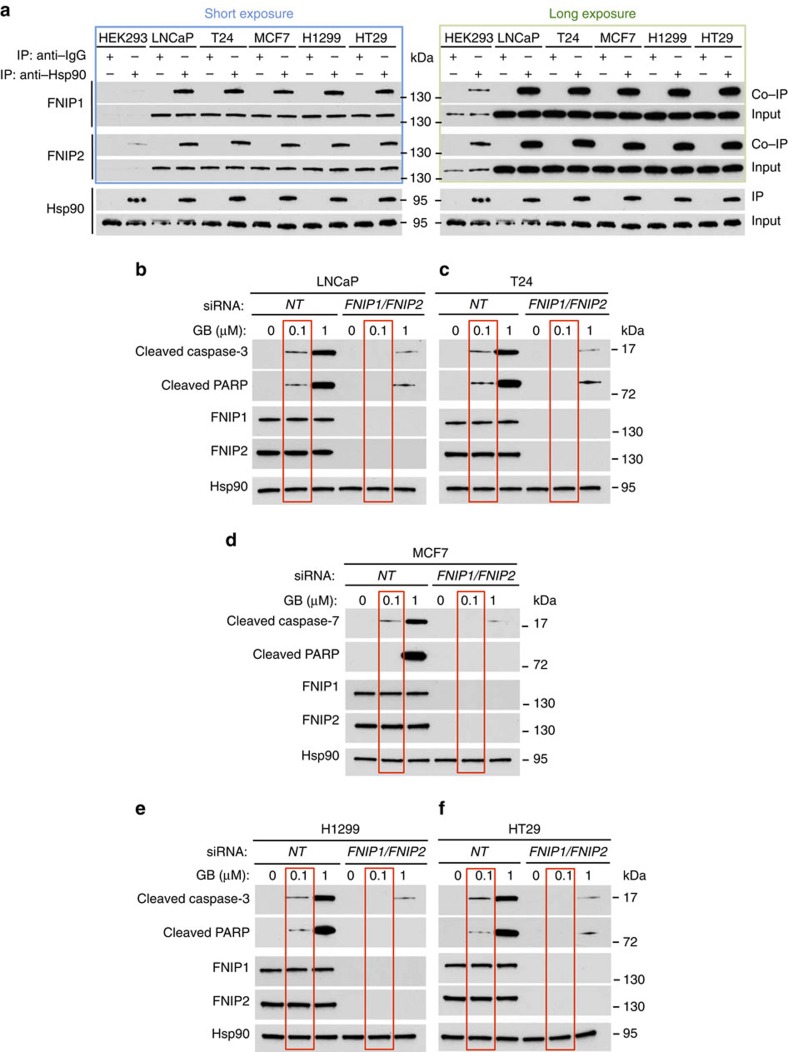
FNIPs expression sensitizes cancer cells to Hsp90 inhibitor. (**a**) Hsp90 was immunoprecipitated from HEK293 cells and LNCaP (prostate), T24 (bladder), MCF7 (breast), H1299 (lung) and HT29 (colorectal) cancer cell lysates using anti-Hsp90 or IgG (control), and immunoblotted with FNIP1 and FNIP2 antibodies to confirm protein interaction. Short and long exposures of the blots are shown. (**b**) Both *FNIP1* and *FNIP2* were silenced by siRNA in LNCaP, (**c**) T24, (**d**) MCF7, (**e**) H1299 and (**f**) HT29, and then treated with indicated amounts of GB. Apoptotic markers were shown by immunoblotting using anti-cleaved caspase-3 and cleaved PARP antibodies. Anti-cleaved caspase-7 antibody was only used for MCF7 cells.

Tumours are generally sensitive to Hsp90 inhibitors^19^,^21^,^22^. We therefore asked whether FNIPs are overexpressed in cancer cells and also whether they are contributing to cancer cell sensitivity towards Hsp90 inhibitors. We used prostate (LNCaP), bladder (T24), breast (MCF7), lung (H1299) and colorectal adenocarcinoma (HT29) cancer cell lines. Previous work by Synta Pharmaceuticals Corp. (data available at www.syntapharma.com/) has established these cell lines' sensitivity to Hsp90 inhibitor GB and these are: LNCaP (IC50=8 nM), T24 (IC50=7 nM), MCF7 (IC50=25 nM), H1299 (IC50=6 nM) and HT29 (IC50=50 nM). We first showed that FNIPs were highly expressed in these cancer cells compared with HEK293 cells (Fig. 9a). We next immunoprecipitated the endogenous Hsp90 from the above cancer cells and detected its interaction with FNIPs (Fig. 9a). To link the high levels of FNIPs with sensitivity of cancer cells to Hsp90 inhibitors, we used siRNA to knock down *FNIP1* and *FNIP2* in LNCaP, T24, MCF7, H1299 and HT29 cells. The apoptotic markers cleaved caspase-3 and cleaved PARP were abundant in cancer cells treated with 0.1 μM GB; however, this effect was abrogated in the siRNA FNIPs knockdown samples (Fig. 9b–f). These data suggest that overexpression of FNIPs is a contributing factor towards cancer cell sensitivity to Hsp90 inhibitor GB. It is noteworthy that cleaved caspase-7 was used to evaluate apoptosis in MCF7 cells, as they lack caspase-3 (Fig. 9d).

### Elevated FNIPs sensitize renal tumours to Hsp90 inhibitor

**Figure 10:**
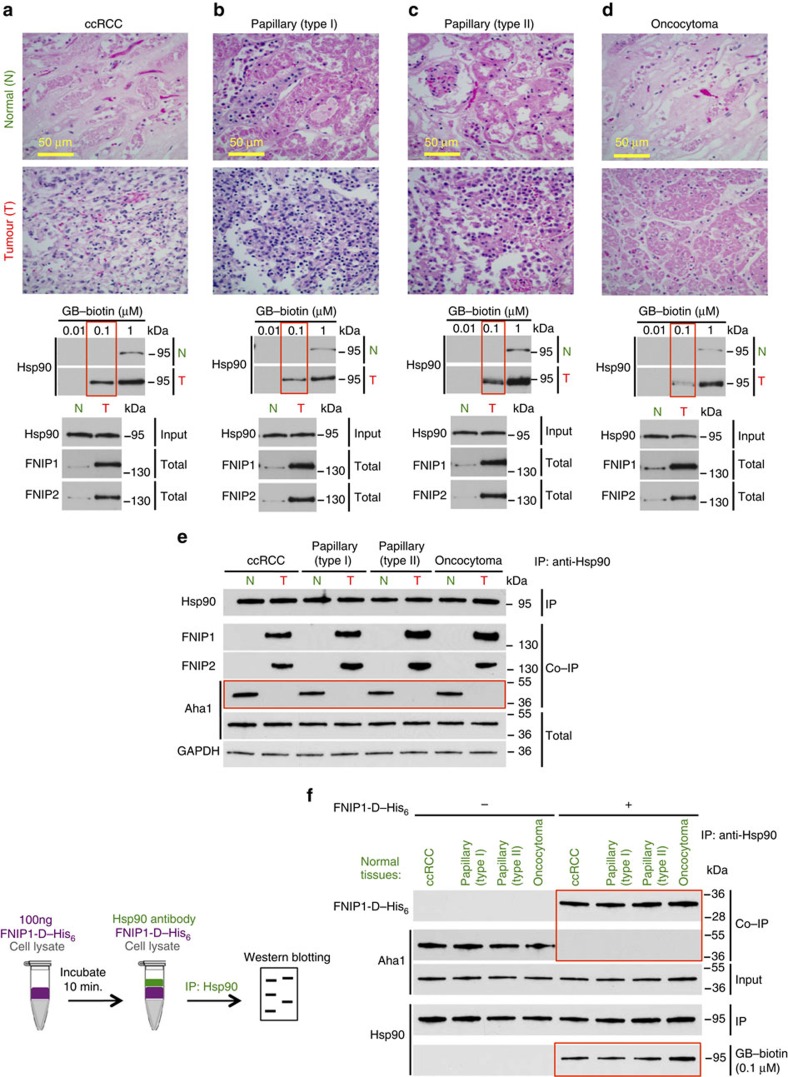
High levels of FNIPs make renal tumours sensitive to Hsp90 inhibitor GB. (**a**) Clear cell renal cell carcinoma (ccRCC), (**b**) Papillary type I, (**c**) Papillary type II, (**d**) Oncocytoma (Tumours, T) and adjacent normal tissues (Normal, N) were stained with haematoxylin and eosin (H&E). Proteins were also extracted from these tumours and adjacent normal tissues and incubated with indicated amounts of biotinylated GB followed by streptavidin agarose beads. Hsp90 was detected by immunoblotting. Expression of FNIP1 and FNIP2 in these samples was also detected by immunoblotting. (**e**) Hsp90 immunoprecipitated from tumours (T) and adjacent normal tissues (N) in **a**–**d**. Co-IP of FNIPs and Aha1 was examined by immunoblotting. (**f**) Lysates from normal tissues in **a**–**d** were incubated with or without 100 ng of pure FNIP1-D–His_6_ for 10 min. Hsp90 was immunoprecipitated and co-IP of FNIP1-D–His_6_ and Aha1 were shown by immunoblotting.

To gain further insight into FNIPs expression and sensitivity of tumours to Hsp90 inhibitors, we examined tumours and adjacent normal tissues from patients with renal cell carcinoma (RCC), the most common type of kidney cancer^23^. Histopathologically, RCCs are classified into subtypes. We used tumours from patients with clear cell RCC, papillary type I and type II RCC, and oncocytoma (Fig. 10a–d). Within 10 min of removal of tumours, by radical or partial nephrectomy, RCC tumours and adjacent normal tissues were dissected into 3 mm^3^ pieces followed by protein extraction and drug binding assay. Our data showed that both FNIP1 and FNIP2 were overexpressed in RCC tumours compared with adjacent normal tissues (Fig. 10a–d). In addition, we discovered that the Hsp90 from RCC tumours had a higher affinity for binding to biotinylated GB compared with the adjacent normal tissues (Fig. 10a–d). We next showed greater association between Hsp90 and FNIPs compared with normal tissues (Fig. 10e). This is not surprising, because FNIPs are highly expressed in tumours compared with adjacent normal tissues. However, we found that Hsp90 bound stronger to Aha1 in normal tissues compared with the tumours, even though both tissues expressed equal levels of Aha1 (Fig. 10e). Our earlier data showed that FNIPs compete with the activating co-chaperone Aha1 for binding to Hsp90 (Fig. 6e). We therefore asked whether addition of purified FNIP1-D–His_6_ to the protein lysates from normal tissues could disrupt the Hsp90–Aha1 complex. Addition of 100 ng FNIP1-D–His_6_ to protein lysates from normal tissues displaced Aha1 interaction with Hsp90 and also increased Hsp90 binding to 0.1 μM biotinylated GB (Fig. 10f). Taken together, our data suggest that FNIPs make renal tumours sensitive to Hsp90 inhibitors. Their expression level can potentially serve as a predictive indicator of tumour response to Hsp90 inhibitors.

## Discussion

Germline mutation in the tumour suppressor *FLCN* is responsible for Birt–Hogg–Dubé syndrome, an inherited kidney cancer syndrome^24^. Previous work has shown that FNIP1 and FNIP2 are critical components of the FLCN complex and are essential for its tumour suppressive function^9^. However, the exact molecular function of FNIP1 and FNIP2 remains elusive. In this study, we demonstrate by MS analysis that FLCN interacts with the molecular chaperones Hsp70 and Hsp90, as well as members of the chaperonin system TRiC. Our data have shown that FLCN is a *bona fide* Hsp70 and Hsp90 client, as treating the cells with the Hsp70 inhibitor JG-98 destabilizes FLCN, and furthermore Hsp90 inhibitor treatment GB, SNX2112 or PU-H71 leads to dissociation of FLCN from Hsp90 and consequently its ubiquitination and proteasomal degradation. Based on our current knowledge in the function of TRiC–Hsp70–Hsp90 proteins and also our findings here, we would like to suggest that TRiC-Hsp70 are involved in ‘early' stages of FLCN folding and Hsp90 plays a key role in protecting FLCN from degradation. We further demonstrated that FLCN binding partners FNIP1 and FNIP2 function as co-chaperones of Hsp90. FNIP1 interacts with the M- and C- domains of Hsp90, whereas FNIP2 interacts only with its M- domain. Previous work reported that FNIP1 and FNIP2 exist as a homodimer and a heterodimer, respectively^10^. We confirmed these data by demonstrating the existence of a FNIP1:FNIP2 heterodimer and its interaction with FLCN. We have also demonstrated that three populations of FNIP1 and FNIP2 interact with Hsp90; however, the FNIP1 homodimer preferentially binds to Hsp90. Our *in vitro* data provide additional evidence that recombinant FNIP1 directly interacts with Hsp90; however, FLCN requires the presence of FNIP1 for its binding to Hsp90. These data suggest that FNIP1 and FNIP2, similar to the co-chaperone Cdc37, are involved in ‘loading' of FLCN and perhaps other client proteins to Hsp90. Approximately 93% of all pathogenic FLCN mutations prematurely truncate the encoded FLCN protein^25^. When experimentally modelled *in vitro*, most mutations analysed appeared to be pathogenic by disrupting stability of FLCN^25^. In addition, FLCN binding to both FNIP1 and FNIP2 is mediated specifically through the C-terminal region of FLCN^8^,^26^. Truncating mutations result in loss of the C-terminus of FLCN, abolishing its interaction with FNIP1 and FNIP2, and consequently Hsp90. We speculate that loss of Hsp90 interaction with FLCN is the reason for the instability of the pathogenic FLCN mutants.

Hsp90 chaperone activity is coupled to its ATPase activity, which is tightly regulated by co-chaperones and posttranslational modification^27^,^28^. Our results suggest that FNIP1 is a potent inhibitor of Hsp90 ATPase activity, as 200 nM of FNIP1 inhibits Hsp90 ATPase activity by 50-fold. FNIP2 also has shown inhibitory activity towards Hsp90; however, it required 1.6 μM of FNIP2 to inhibit the ATPase activity by eightfold. Although we use the term ‘inhibition' here, FNIPs seem only to be slowing the chaperone cycle. Their effect on Hsp90 is not similar to the small molecule inhibitors of Hsp90 that bind to the N-domain and inhibit Hsp90 chaperone function. The reason for this conclusion stems from our observation that overexpression of FNIP1 or FNIP2 does not lead to degradation of Hsp90 client proteins. In addition, chaperoning of the proteins such as CFTR that require a slow chaperone cycle was enhanced by overexpression of FNIP1 and FNIP2. We have also shown that overexpression of FNIPs enhance Hsp90 binding to GB and SNX2112. Moreover, Hsp90 from kidney tumours bound stronger to GB compared with Hsp90 from adjacent normal tissues. This observation correlated with the overexpression of both FNIP1 and FNIP2 in tumours compared with adjacent normal tissues. Our findings suggest that FNIPs expression level can potentially serve as a predictive indicator of tumour response to Hsp90 inhibitors.

What is the relationship between FNIPs and Aha1 towards binding to Hsp90? FNIPs bind to the M-domain of Hsp90 and may interfere with the N-domain interaction with the catalytic domain. We also found that FNIPs compete with Aha1 co-chaperone binding to Hsp90. Our data have demonstrated the ability of FNIP1 and Aha1 to compete for binding to Hsp90, fine tuning its chaperone cycle. The competition between Aha1 and FNIPs binding to Hsp90 was also observed in renal tumours and their adjacent normal tissues. We found that Aha1 bound stronger to Hsp90 in normal tissues; however, overexpression of FNIPs appears to displace Aha1 from Hsp90 and increase Hsp90 binding to its inhibitors. Our data are in agreement with previously published work by Workman's laboratory^29^, suggesting that Aha1 dissociation from Hsp90 plays a role in drug sensitivity. What determines the binding of these proteins to Hsp90? We have already shown that posttranslational modifications of Hsp90 determine its binding to co-chaperones^28^. In addition, our recent work has shown that c-Abl-mediated phosphorylation of Y223-Aha1 promotes its interaction with Hsp90 (ref. 19). We therefore suspect similar mechanisms exist between these three proteins, where cross-talk between posttranslational modifications is a determining factor for differential binding of Aha1 and FNIPs to Hsp90.

## Methods

### Plasmids and yeast strains

FLCN complementary DNA was synthesized in FLAG-tagged pcDNA3.1 vector backbone (Thermo Fisher Scientific) by GeneWiz, Inc. FNIP1–His_6_ and FNIP2–His_6_ messenger RNA was also synthesized in pcDNA3.1 by GeneWiz, Inc. and subcloned into pCMV-Myc and pCMV–HA vectors (ClonTech). The EVs containing these tags were used and also referred to as controls. For bacterial expression of FNIP1, FNIP2, FNIP1-D and Hsp90α, pRSET-A expression vector containing a 6 × His tag (ThermoFisher Scientific) was used. The HA–FNIP1 domain-specific plasmids were previously reported^8^. Hsp90α domain-specific plasmids were amplified using primers listed in Supplementary Table 3. FNIP1–FLAG and FNIP2–FLAG were cloned in to yeast expression plasmid pYES2 using primers FNIP1–KpnI–FLAG–F/FNIP1–XhoI–R and FNIP2–KpnI–FLAG–F/FNIP2–XhoI–R.

### Yeast growth media

Yeast cells were grown on YPDA (2% (w/v) Bacto peptone, 1% yeast extract, 2% glucose and 20 mg per litre adenine) and YPGal (2% (w/v) Bacto peptone, 1% yeast extract, 2% galactose and 20 mg per litre adenine). Selective growth was on dropout 2% glucose (DO) medium with appropriate amino acids^30^. Medium pH was adjusted to 6.8 with NaOH before autoclaving. The yeast strain pp30 (*MAT* a, *trp1-289*, *leu2-3*,*112*, *his3-200*, *ura3-52*, *ade2-101*, *lys2-801*, *hsc82KANMX4* and *hsp82KANMX4*) expressing Hsp82-Ycplac111 or Hsp90α-Ycplac111 as the sole Hsp90 were used in this study. These yeast strains were reported previously by Mollapour *et al*.^31^. Glutathione *S*-transferase (GST)-tagged Lst4 under control of the GAL1/10 promoter expressed in a BY4741 (MATa) background was acquired from GE Dharmacon.

### Mammalian cell culture

Cultured cell lines human embryonic kidney (HEK293) grown in DMEM (Sigma-Aldrich), human bladder (T24) and human colorectal adenocarcinoma (HT29) grown in McCoy's 5a medium (Sigma-Aldrich), human prostate cancer (LNCaP) and human non-small cell lung cancer (H1299) grown in RPMI 1640 medium (Sigma-Aldrich) and human breast adenocarcinoma (MCF7) grown in Eagle's minimum essential medium (Sigma-Aldrich). Cultured cell lines were acquired from (American Type Culture Collection) and their media was supplemented with 10% fetal bovine serum (Sigma-Aldrich) and grown in a CellQ incubator (Panasonic Healthcare) at 37 **°**C in an atmosphere containing 5% CO_2_.

### *Ex vivo* culture and analysis of human RCC tumours

Tumour and adjacent normal tissues of the patients with conventional RCC were obtained with written informed consent from the Department of Urology at SUNY Upstate Medical University. At the time of radical or partial nephrectomy, which was done with <10 min of renal ischaemia, RCC tumours were dissected into ∼3 mm^3^ pieces and protein was extracted and quantified as previously described in detail^22^.

### Bacterial expression and purification of proteins

All proteins were expressed in *Escherichia coli* strain BL21 (DE3) and included an N-terminal 6 × His tag. Purification buffers included 20–50 mM Tris or phosphate pH 8.0 and 10 mM β-mercaptoethanol. Chromatography resins were purchased from GE Healthcare Bio-Sciences (Marlborough, MA), except for Ni-NTA agarose, which was purchased from Qiagen (Valencia, CA). Transformed cells were grown at 37 °C in lysogeny broth (LB) with 50 mg l^−1^ ampicillin until OD_600_=0.6. For Hsp90α, cultures were then cooled to 20 °C and induced with 20 mg l^−1^ isopropyl-β-D-thiogalactoside overnight. Cells were harvested by centrifugation and lysed enzymatically. Hsp90α expressed in the supernatant and was isolated by sequential Ni-NTA metal affinity (10–250 mM imidazole step gradient), Q-Sepharose anion exchange (0–1 M NaCl gradient) and Superdex-75 size-exclusion chromatography. Purified Hsp90α was nucleotide free evidenced by an *A*_280/260_ ratio of 1.83. For FNIP1-D, cells were induced with 20 mgl ^−1^ isopropyl-β-D-thiogalactoside at 37 °C for 3 h, harvested by centrifugation and lysed by sonication. FNIP1-D expressed in inclusion bodies and was purified by washing the pellet 3 × in buffer, dissolving the pellet in 8 M urea, subjecting the dissolved pellet to Ni-NTA metal affinity chromatography (10–250 mM imidazole step gradient) and refolding by rapid dilution. Proteins were >90% pure. Concentrations were determined using calculated extinction coefficients as previously described^19^. Proteins were flash frozen on dry ice and stored at −80°C until use.

### Immunoprecipitation and immunoblotting

For IP, mammalian cell lysates were incubated with anti-FLAG antibody conjugated beads (Sigma) for 2 h at 4 °C. Pulldowns were achieved by incubating lysate with Ni-NTA agarose (Qiagen) for 2 h at 4 °C or with anti-Hsp90 antibody (835-16F1, Enzo Life Sciences), or anti-Hsp82 (yHsp90), FLCN (Cell Signaling), FNIP1 or FNIP2 (NCI) for 1 h followed by protein G agarose for 2 h at 4 °C. Immunopellets were washed four times with fresh lysis buffer (20 mM HEPES pH 7.0, 100 mM NaCl, 1 mM MgCl_2_, 0.1% NP40, protease inhibitor cocktail (Roche) and PhosSTOP (Roche). Proteins bound to Ni-NTA agarose were washed with 50 mM imidazole in lysis buffer (20 mM Tris-HCl pH 7.5, 100 mM NaCl, protease inhibitor cocktail (Roche) and PhosSTOP (Roche)) and eluted with either 300 mM imidazole in lysis buffer or with 5 × Laemmli buffer. Precipitated proteins were separated by SDS–PAGE and transferred to nitrocellulose membranes. Co-immunoprecipitated proteins were detected by immunoblotting with indicated dilutions of antibodies recognizing 1:8,000 FLAG (Cat. A2220, Sigma-Aldrich); 1:2,000 Tetra-His (Cat. 34670, Qiagen); 1:8,000 Hsp90-835-16F1 (Cat. ADI-SPA-835), 1:10,000 GAPDH (Cat. ADI-CSA-335), 1:2,000 p23 (Cat. ADI-SPA-670) (ENZO Life Sciences); 1:20,000 Hsp70 (Cat. SPC-103C/D, StressMarq); 1:1,000 CFTR (Cat. 05-583, Millipore); 1:4,000 FLCN (Cat. 3697), 1:2,000 GR (Cat. 12041), 1:2,000 p-GR-S211 (Cat. 4161), 1:2,000 ERα (Cat. 13258), 1:1,000 CHIP (Cat. 2080), 1:2,000 Grp94 (Cat. 2104), 1:2,000 cMyc (Cat. 2276), 1:2,000 HA (Cat. 3724), 1:1,000 CCT2 (Cat. 3661), 1:1,000 ULK1 (Cat. 8054), 1:1,000 PP5 (Cat. 2289), p50^cdc37^ (Cat. 4793), 1:2,000 c-Src (Cat. 2123), 1:2,000 p-c-Src-Y416 (Cat. 2101), 1:4,000 Akt (Cat. 2967), 1:2,000 p-Akt-S473 (Cat. 4060) and 1:2,000 p60^Hop^ (Cat. 5670), cleaved caspase-3 (Asp175) (Cat. 9664), 1:2,000 cleaved caspase-7 (Asp198) (Cat. 9491) and 1:2,000 cleaved PARP (Asp214) (Cat. 9544) (Cell Signalling); 1:4,000 GST (Cat. 374171), 1:4,000 Raf-1 (Cat. sc-133), 1:4,000 Cdk4 (Cat. sc-601) and 1:1,000 Ubiquitin (Cat. sc-8017) (Santa Cruz Biotechnology); and 1:1,000 Aha1 (Cat. 600-401-974, Rockland). Antibodies (1:500 FNIP1 and 1:500 FNIP2) were generated at NCI-NIH. Sba1 and Hsp82 (yHsp90) antibodies were generated at Institute of Cancer Research, UK. Secondary antibodies raised against goat (Cat. sc-2020), mouse (Cat. sc-2005), rabbit (Cat. sc-2004) and rat (Cat. sc-2006) (Santa Cruz Biotechnology) were used at 1:4,000 dilution. Representative uncropped western blottings are shown in Supplementary Fig. 6.

### Protein labelling and *K*_d_ measurements

FNIP1-D protein was desalted into 50 mM Tris pH 7.2, 150 mM NaCl and 1 mM TCEP using a PD-10 desalting column and labelled with Texas Red C2 maleimide. Labelling stoichiometry was 0.75 labels/FNIP1-D using *ɛ*_595_=104,000 M^−1^ cm^−1^ for Texas Red and a correction factor of 0.26 × *ɛ*_595_ to account for its absorbance at 280 nm. Hsp90α at the indicated concentrations was incubated on ice in 50 mM Tris pH 7.2, 150 mM NaCl, 1 mM TCEP, 4 mM MgCl_2_ with or without 1 mM ATP, ADP or AMPPNP for 10 min, then incubated with 1 μM labelled FNIP1-D for 30 min. Fluorescence anisotropy was measured using a SpectraMax i3 equipped with rhodamine fluorescence polarization module (*λ*_ex_\*λ*_em_=535 nm/595 nm). Curve fitting was done in KaleidaGraph 4.0.

### MS analysis

Visible bands were excised from the gel manually and cut into small pieces ∼1 mm × 1 mm. These gel pieces were destained using 1:1 30-mM potassium ferricyanide:100-mM sodium thiosulfate for 10 min. The destained gel pieces were then washed with 25 mM ammonium bicarbonate and acetonitrile alternatively for 5 min each wash. This cycle of 5 min 25 mM ammonium bicarbonate wash followed by 5 min acetonitrile wash was repeated three times. To further prepare the gel pieces for digestion, the gel pieces were then dehydrated in 100% acetonitrile. After removing all acetonitrile, 25 μl of porcine trypsin (Promega) dissolved in 25 mM ammonium bicarbonate at a concentration of 20 μg ml^−1^ was added to the gel pieces. The gel pieces were then kept at room temperature overnight (∼12–16 h). Following digestion, the supernatant was transferred to a second tube and acetonitrile was added to the gel pieces to complete the extraction of digested peptides. This extract was added to the first supernatant and this combined solution, containing the extracted peptides, was frozen and lyophilized. The peptides were resuspended in 5 μl of 100:99:1 acetonitrile:water:trifluoroacetic acid immediately before spotting on the matrix-assisted laser desorption/ionization (MALDI) target.

For MALDI analysis, the matrix solution consisted of α-cyano-4-hydroxycinnamic acid (Aldrich Chemical Co., Milwaukee, WI) saturating a solution of 1:1:0.01 acetonitrile:25 mM ammonium citrate:trifuoroacetic acid. Approximately 0.15 μl of peptide solution was spotted on the MALDI target immediately followed by 0.15 μl of the matrix solution. This combined solution was allowed to dry at room temperature. MALDI–MS and MS/MS data were then acquired using the ABSCIEX TOF/TOF 5800 Mass Spectrometer. Resultant peptide mass fingerprint and peptide sequence data were submitted to the UniProt database using the Mascot search engine to which relevance is calculated and scores are displayed.

### Hsp90 ATPase activity *in vivo*

ATPase activity of human Hsp90α isolated from prostate cancer PC3 cells and its activation by Aha1 or inhibition by HA–FNIP1, HA–FNIP1-D and HA–FNIP2 from HEK293 cells were measured as previously described^19^. Cells were transiently transfected with Hsp90α–HA (PC3) or the Aha1–FLAG, HA–FNIP1, HA–FNIP1-D and HA–FNIP2 constructs (HEK293). Following protein extraction and IP, protein-bound HA (Sigma) or FLAG (Sigma) affinity beads were washed five times in 0.5 M NaCl and 1% NP40 buffer. Proteins were competed off the beads with either HA or FLAG (Sigma) peptide, at 4 °C for 2 h with agitation. Protein was then concentrated with Amicon Ultra-2 ml, 10 K centrifugal filters (Millipore). Using the Micro BCA Protein Assay Kit (Thermo Scientific), protein was quantified and 1 μg was run on an SDS–PAGE gel as described previously, to standardize the amount of protein used in the assay. Assay was performed as described in the P_i_Per Phosphate Assay Kit instructions for use (Life Technologies). Standard curve with linear fit line was created from 0 to 100 μM final concentration reactions. Hsp90α (2.5 μg) and 5 μg of Aha1, FNIP1, FNIP1-D and FNIP2 were added to each reaction run in triplicate, incubated at 37 °C for 1 h, with 1 mM ATP as substrate and 10 μM GB was used in drug reactions. ATP turnover was calculated as mmol P_i_ per mol Hsp90α per minute and relative ATPase activity was calculated from those values, with the value of Hsp90α alone representing 100% activity.

### Statistical analysis

The data presented are the representative or examples of three biological replicates, unless it is specified. Data were analysed with unpaired *t*-test. Asterisks in figures indicate significant differences (*P<0.05, **P<0.005, ***P<0.0005 and ****P<0.0001). Error bars represent the s.d. or s.e. for three independent experiments, unless it is indicated.

### Data availability

The authors declare that the data supporting the findings of this study are available within the article and its Supplementary Information files.

## Additional information

**How to cite this article:** Woodford, M. R. *et al*. The FNIP co-chaperones decelerate the Hsp90 chaperone cycle and enhance drug binding. *Nat. Commun.* 7:12037 doi: 10.1038/ncomms12037 (2016).

## Supplementary Material

Supplementary Information Supplementary Figures 1-6 and Supplementary Tables 1-3
