# Supplementary material for: The FNIP co-chaperones decelerate the Hsp90 chaperone cycle and enhance drug binding
Source: Nat Commun. 2016 Jun 29;7:12037. doi: 10.1038/ncomms12037 (PMC4931344; doi:10.1038/ncomms12037)
Supplement: Supplementary Information — Supplementary Figures 1-6 and Supplementary Tables 1-3 [file ncomms12037-s1.pdf]

Supplementary Fig. 1

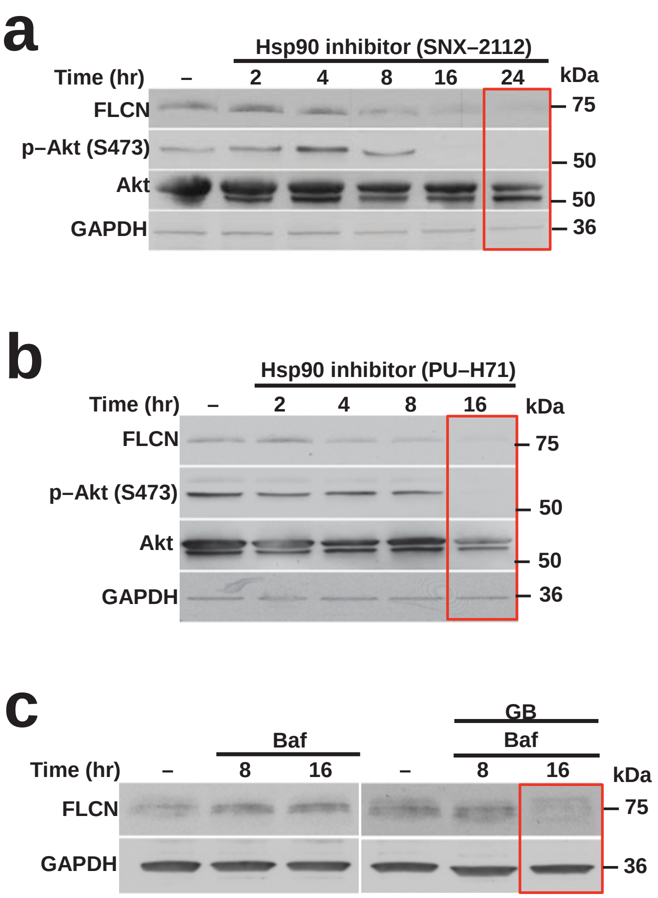

Supplementary Fig. 1. FLCN is a new client of Hsp90. Related to Fig. 1.

a) HEK293 cells were treated with 2μM of the Hsp90 inhibitor SNX–2112 for the indicated times. FLCN protein was detected by immunoblotting.
b) HEK293 cells were treated with 3μM of the Hsp90 inhibitor PU–H71 for the indicated times. FLCN was detected by immunoblotting.
c) 100nM treatment of HEK293 cells with the lysosome inhibitor bafilomycin (Baf) for 8 or 16 hr in presence or absence of 1μM ganetespib (GB).

Supplementary Fig. 2

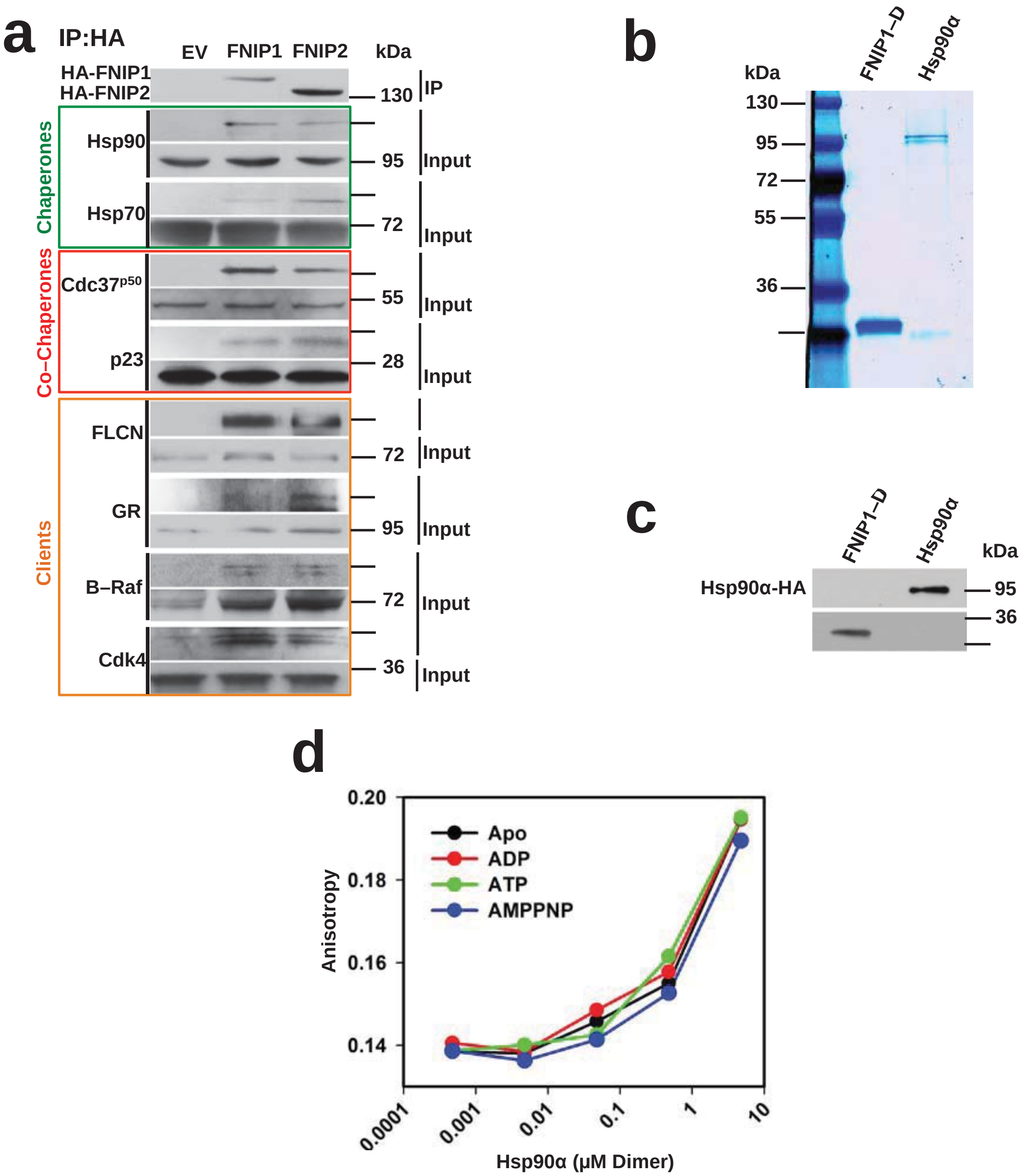

Supplementary Fig. 2. FNIP1 facilitates FLCN binding to the Hsp90 chaperone. Related to Fig. 2-3.

a) Empty vector (EV), HA-FNIP1 or HA-FNIP2 constructs transiently expressed and isolated from HEK293 cells. Interaction of FNIP1 and FNIP2 with the molecular chaperones, co-chaperones and indicated client proteins were assessed by immunoblotting.
b) 100ng of the purified FNIP1-D-His$_6$ and 50ng Hsp90α-HA protein samples were resolved on the SDS-PAGE gel and stained with Coomassie stain.
c) Western blot analysis of the purified proteins from (A).
d) FNIP1-D-His$_6$:HSP90α-HA binding measured by fluorescence anisotropy in the presence and absence of different adenosine nucleotides.

Supplementary Fig. 3

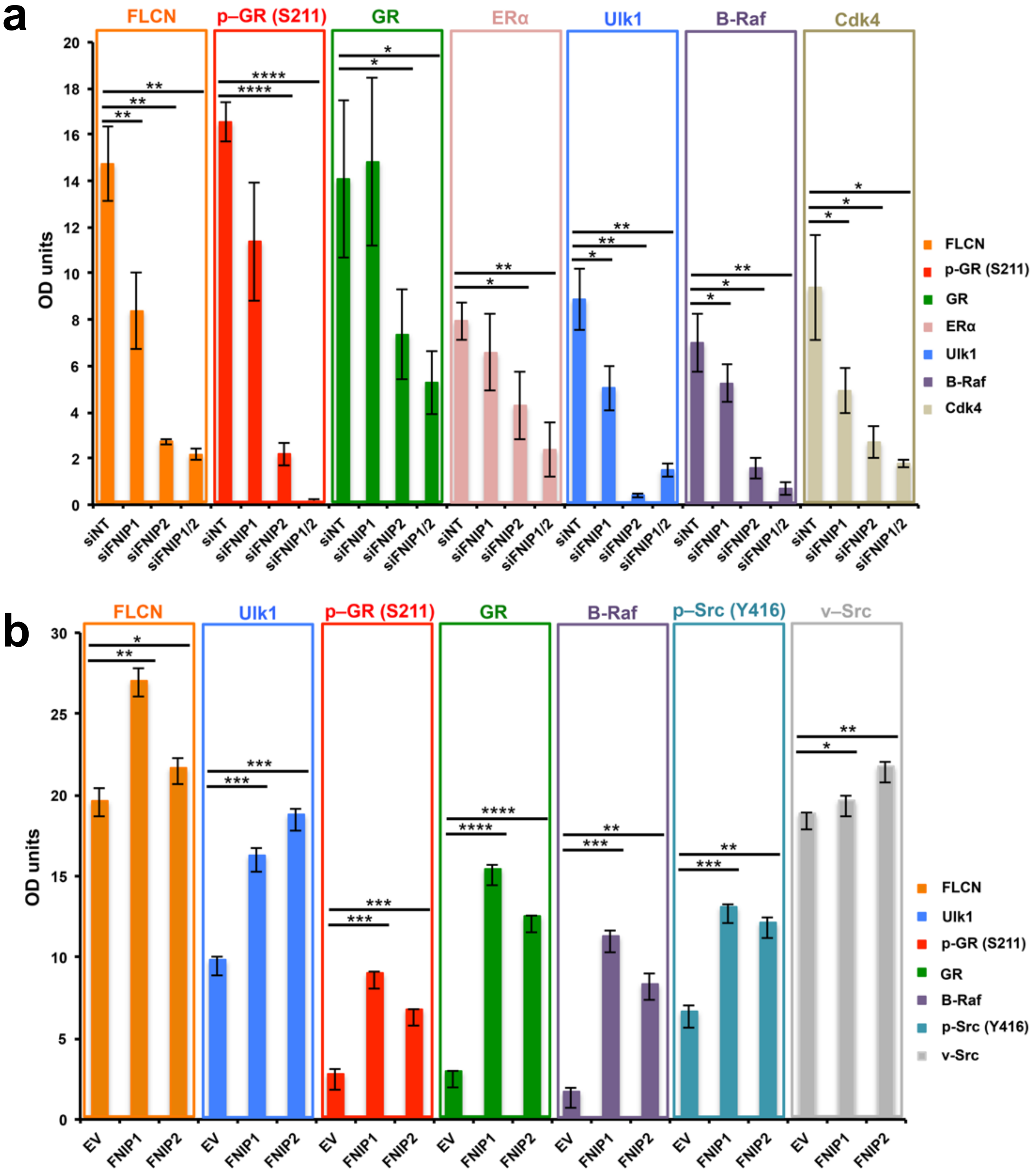

Supplementary Fig. 3 . FNIP co-chaperones facilitate chaperoning of the clients. Related to Fig. 4.

a) Effect of siRNA knockdown of *FNIP1* and *FNIP2* on Hsp90 clients. Stability and activity of the indicated Hsp90 clients were assessed by immunoblotting. Densitometry (optical density, OD) of the Western blot in Fig. 3a are represented as mean ± S.D. (*p < 0.05, **p < 0.005, ****p < 0.00001).

b) Transient overexpression of cMyc-tagged FNIP1 or FNIP2 in HEK293 cells and their impact on levels of Hsp90 clients was assessed by immunoblotting in Fig. 3b. Densitometry (OD) of the Western blot in Fig. 3a are represented as mean ± S.D. (*p < 0.05, **p < 0.005, ***p < 0.0005, ****p < 0.00001).

Supplementary Fig. 4

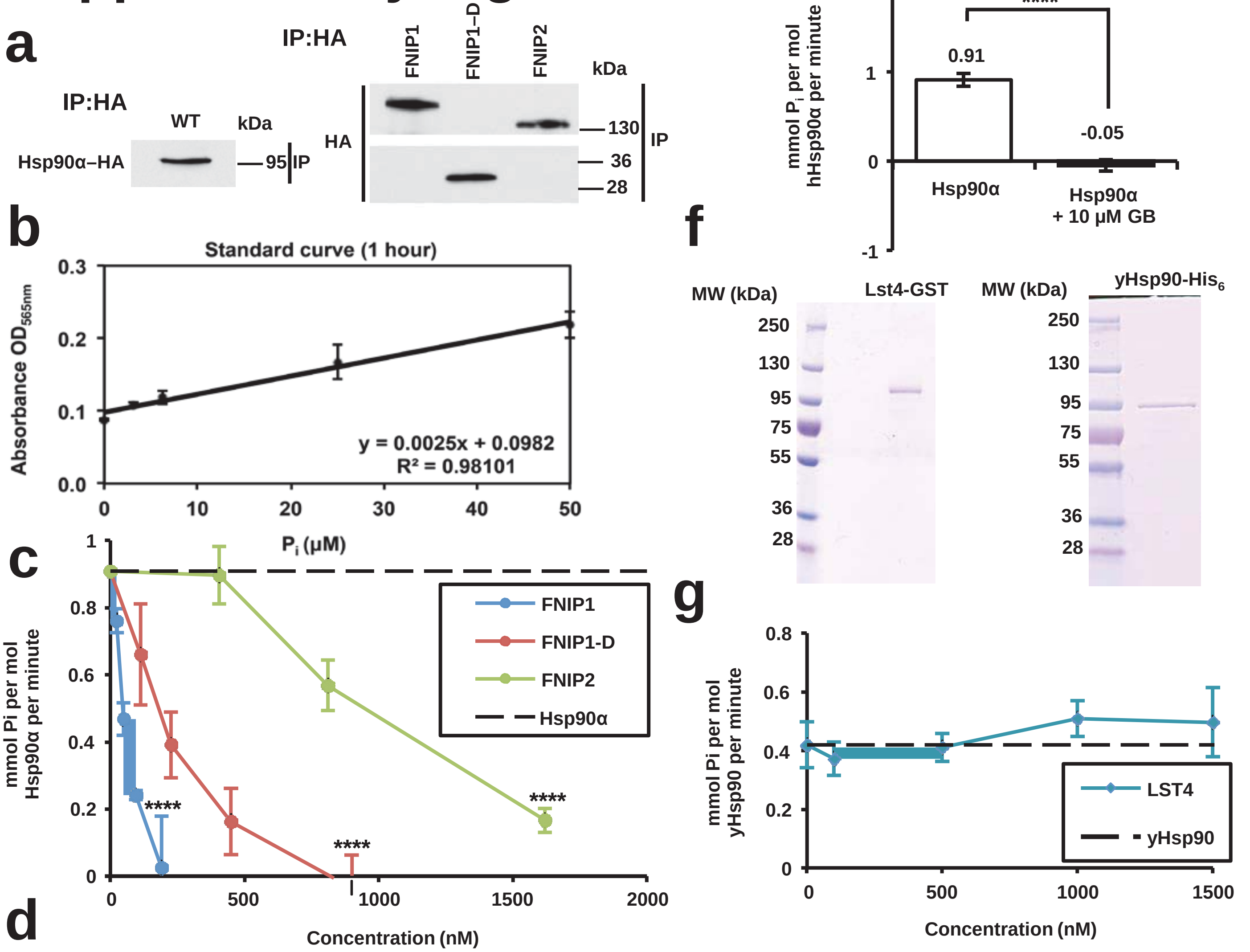

d

Molarity	Ratio to Hsp90α	µg FNIP1
23.72 nM	0.1:1	0.3125
47.44 nM	0.2:1	0.625
94.88 nM	0.3:1	1.25
189.77 nM	0.7:1	2.5

Molarity	Ratio to Hsp90α	µg FNIP1–D
112.41 nM	0.4:1	0.3125
224.82 nM	0.8:1	0.625
449.64 nM	1.5:1	1.25
899.28 nM	3.1:1	2.5

Molarity	Ratio to Hsp90α	µg FNIP2
–	–	–
405.78 nM	1.4:1	5
811.56 nM	3.5:1	10
1623.12 nM	5.6:1	20

Supplementary Fig. 4. FNIP co-chaperones inhibit Hsp90 chaperone cycle and facilitate chaperoning of the clients. Related to Fig. 4-5.

a) PC3 cells were transiently transfected with Hsp90α–HA and HEK293 cells were transiently transfected with HA-FNIP1, HA-FNIP1-D, or HA-FNIP2 constructs. Hsp90α-HA, HA-FNIP1, HA-FNIP1-D, and HA-FNIP2 were immunoprecipitated, competed with the relevant peptides and proteins were detected by immunoblotting.

b) Inorganic phosphate (P_i) standard curve at 1 hr. The x–axis shows µM of P_i per assay and the y–axis shows absorbance at 565nm. Mean ± S.D. from values obtained in three independent experiments.

c) ATPase activity of Hsp90α–HA with or without HA-FNIP1, HA-FNIP1-D, or HA-FNIP2 expressed in mmol P_i per mol Hsp90α per minute. Mean ± S.D. from values obtained in three independent experiments with ***p < 0.0005.

d) Molarity of the proteins in the ATPase assay, ratio to Hsp90, and total quantity of protein added.

e) ATPase activity of Hsp90α–HA with or without 10µM GB expressed in mmol P_i per mol Hsp90α per minute. Mean ± S.D. from values obtained in three independent experiments with ****p < 0.0001.

f) 50ng of Lst4-GST and yHsp90-His6 were resolved on the SDS-PAGE gel and stained with Coomassie stain.

g) *In vitro* ATPase activity of the yHsp90-His_6 with indicated amounts Lst4-GST. All the data represent mean ± S.D.

Supplementary Fig. 5

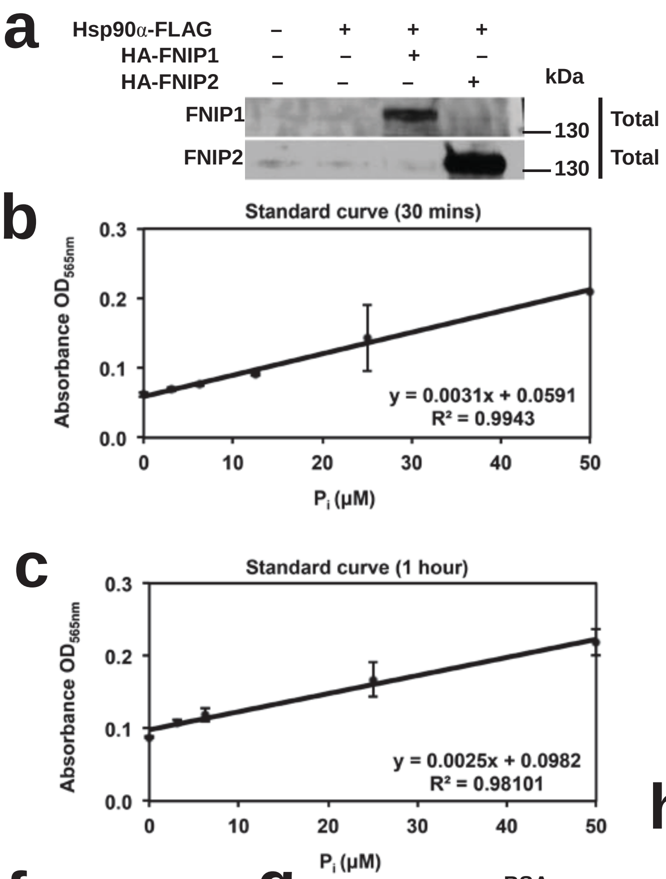

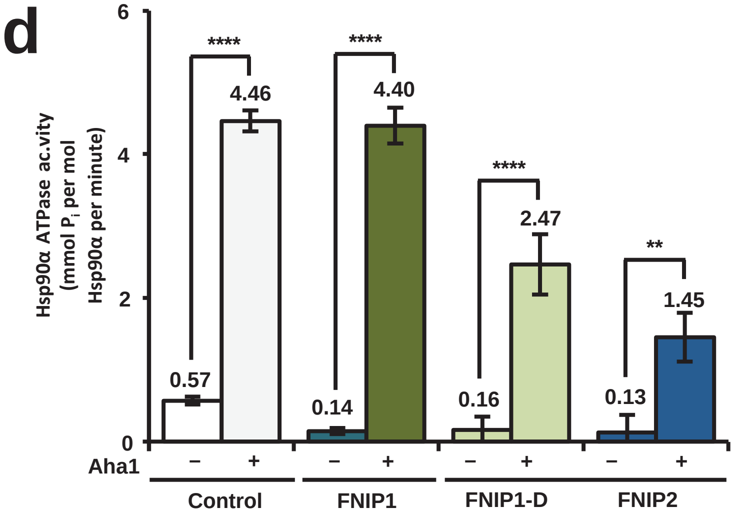

e

Protein	Molarity	Molar ratio to Hsp90	μg protein
Hsp90α	291.5 nM	–	2.5
FNIP1	189.77 nM	0.7:1	2.5
FNIP1–D	899.28 nM	3.1:1	2.5
FNIP2	1.62 μM	5.6:1	20
Aha1	1.27 μM	4.4:1	5

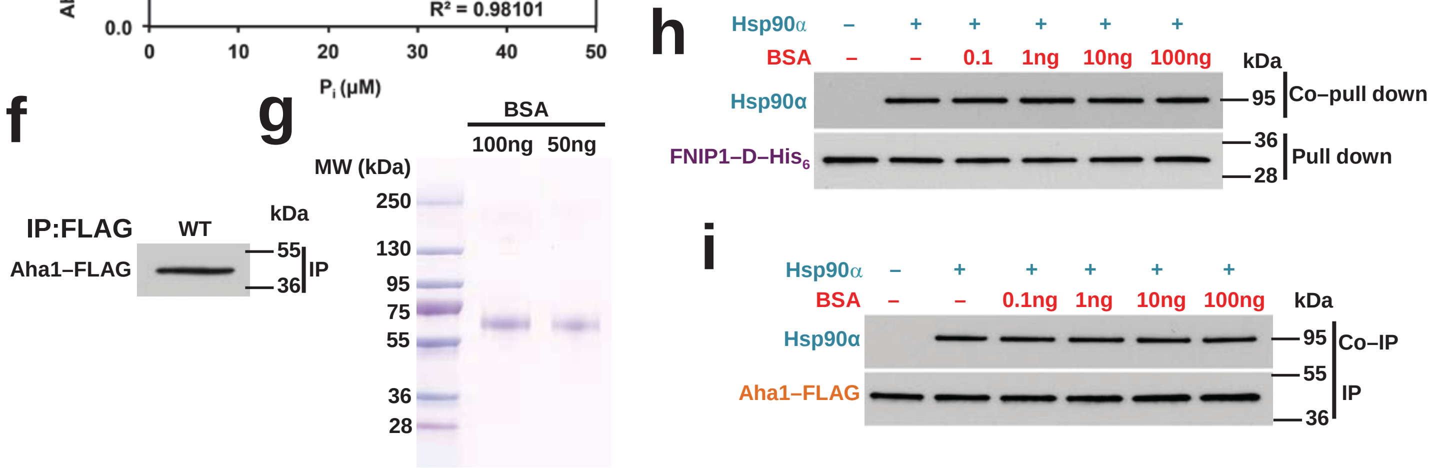

Supplementary Fig. 5. FNIPs compete with the Aha1 co–chaperone for binding to Hsp90. Related to Fig. 6.

a) Hsp90α with and without HA-FNIP1 and HA-FNIP2 proteins from Figure 4B. Western blotting validates overexpression of FNIPs compared to endogenous levels of FNIP1 and FNIP2.
b) Inorganic phosphate (Pi) standard curve at 30 min and
c) 1 hour. The x-axis shows μM of Pi per assay and the y-axis shows absorbance at 565nm. Mean ± S.D. from values obtained in three independent experiments.
d) Inhibition of Hsp90α–HA ATPase activity 30 min after the addition of HA-FNIP1 or HA-FNIP2 and subsequent stimulation by Aha1–FLAG after an additional 30 min expressed in mmol Pi per mol Hsp90α per min. Mean ± S.D. from three independent experiments with $**p < 0.005$, $****p < 0.0001$.
e) Molarity of the protein in the ATPase assay, ratio to Hsp90, and total quantity of protein added are listed.
f) HEK293 cells were transiently transfected with Aha1-FLAG. Following immunoprecipitation, protein was competed with peptide and detected by immunoblotting with anti-FLAG antibody.
g) Indicated amounts of BSA were resolved on the SDS-PAGE gel and stained with Coomassie stain.
h) FNIP1-D-His_6 was attached to Ni-NTA agarose and then incubated with Hsp90α. Ni-NTA agarose was then washed and incubated with the indicated amounts of BSA.
i) Aha1-FLAG attached to anti-FLAG M2 affinity gel was incubated with Hsp90α initially and then washed and incubated with indicated amounts of BSA.

Supplementary Fig. 6. Full original immunoblots and gel images.

Figure 1b

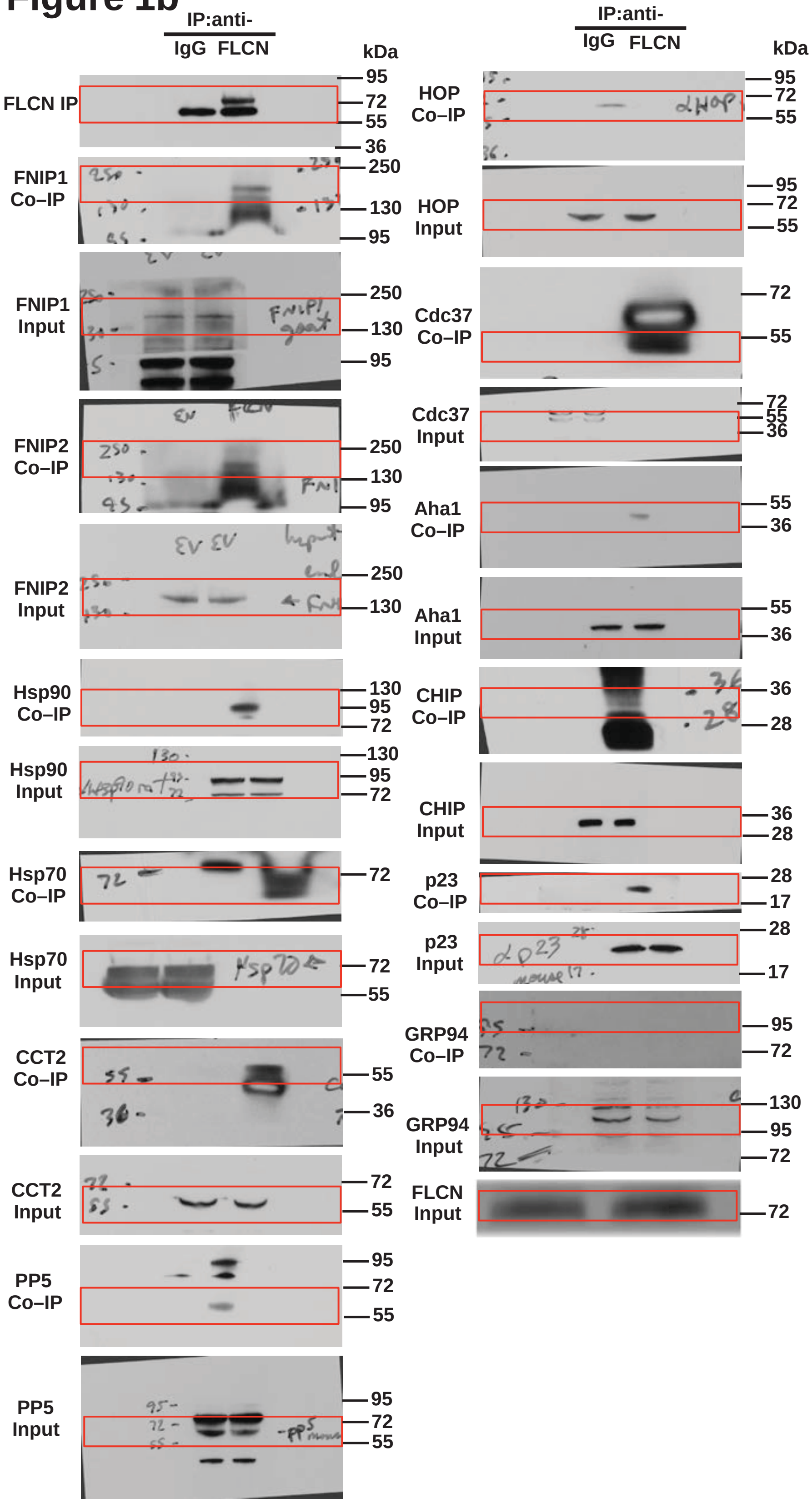

Figure 1c

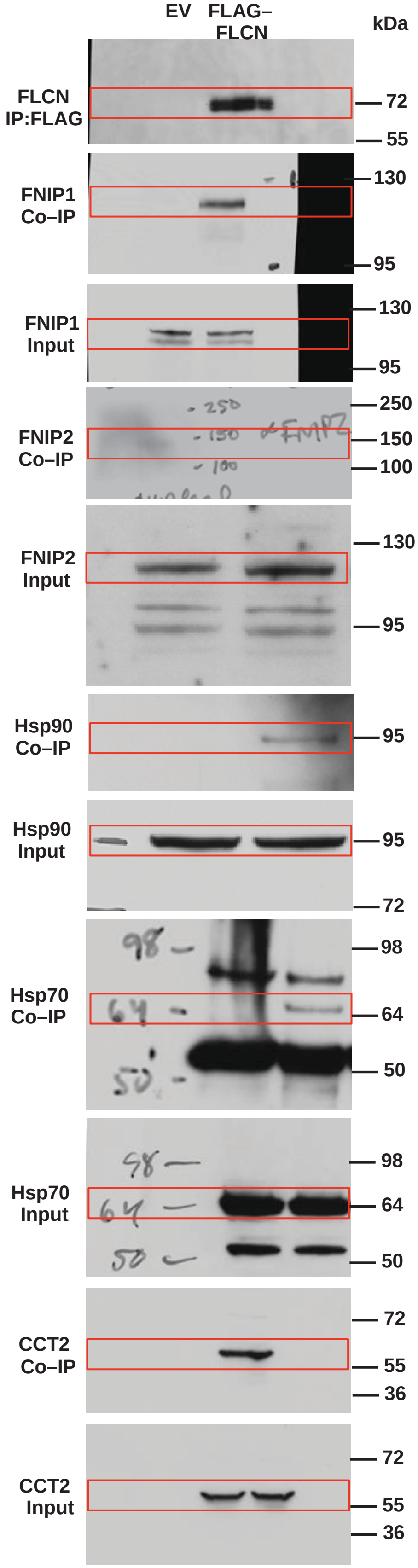

Figure 1c continued

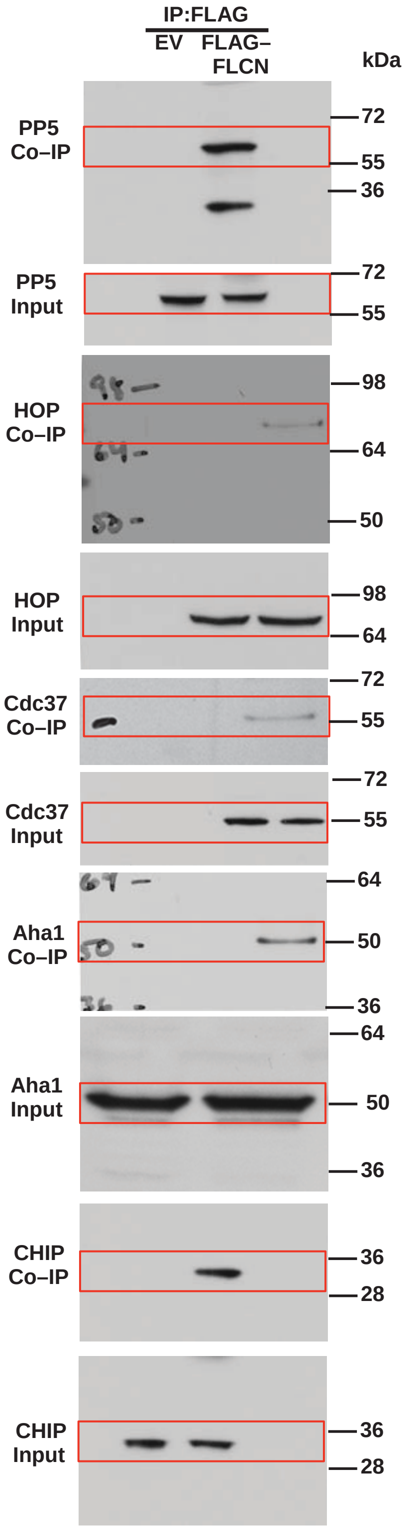

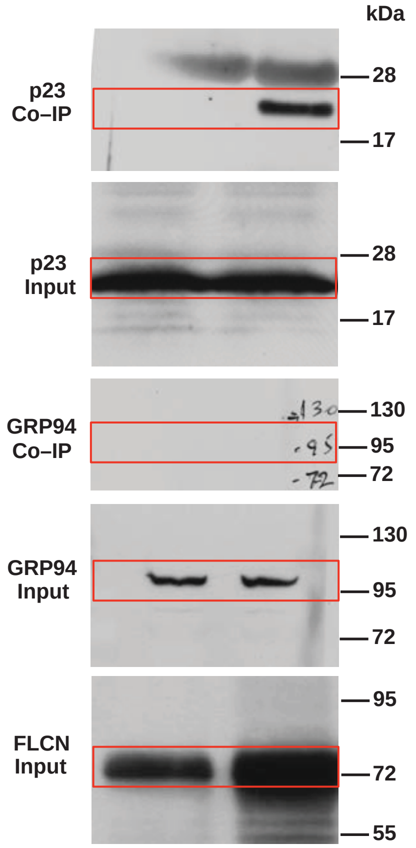

Figure 1e

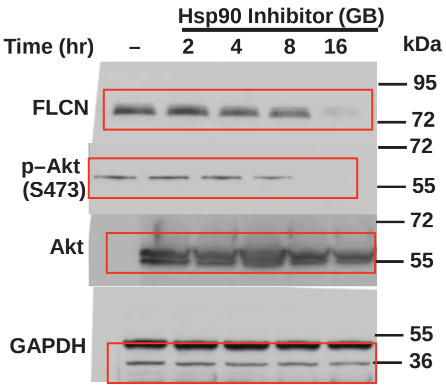

Figure 1f

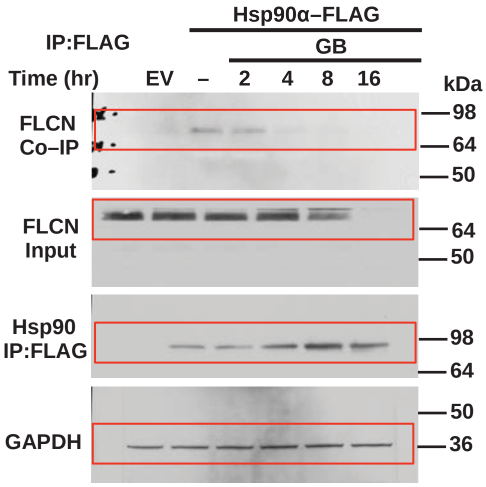

Figure 1d

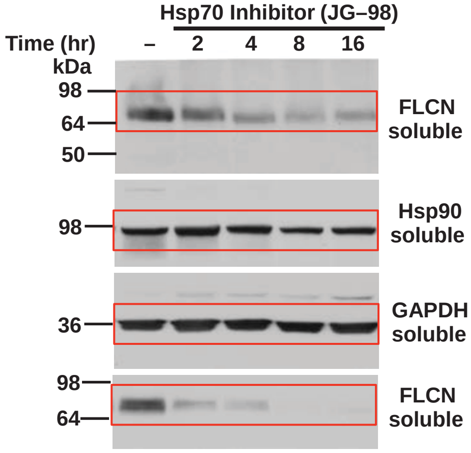

Figure 1g

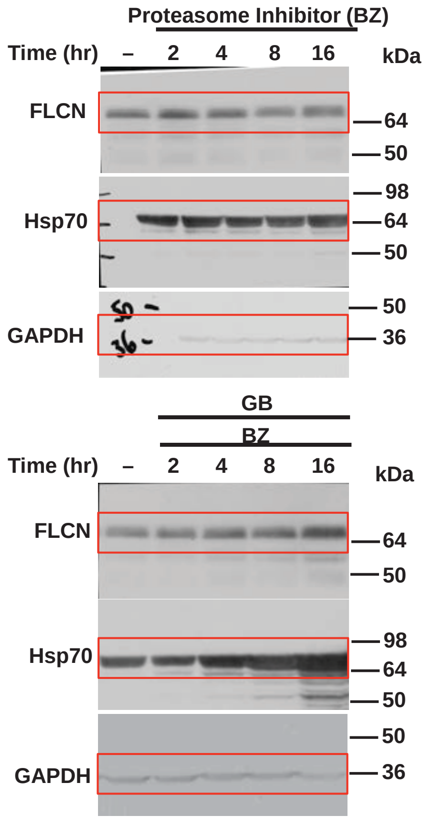

Figure 1h

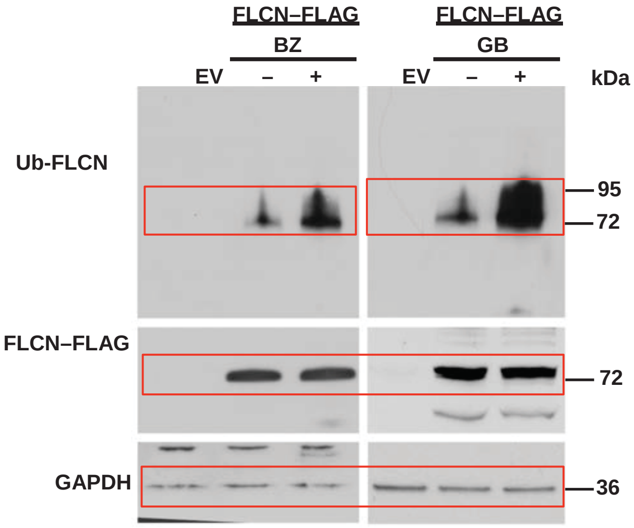

Figure 2a

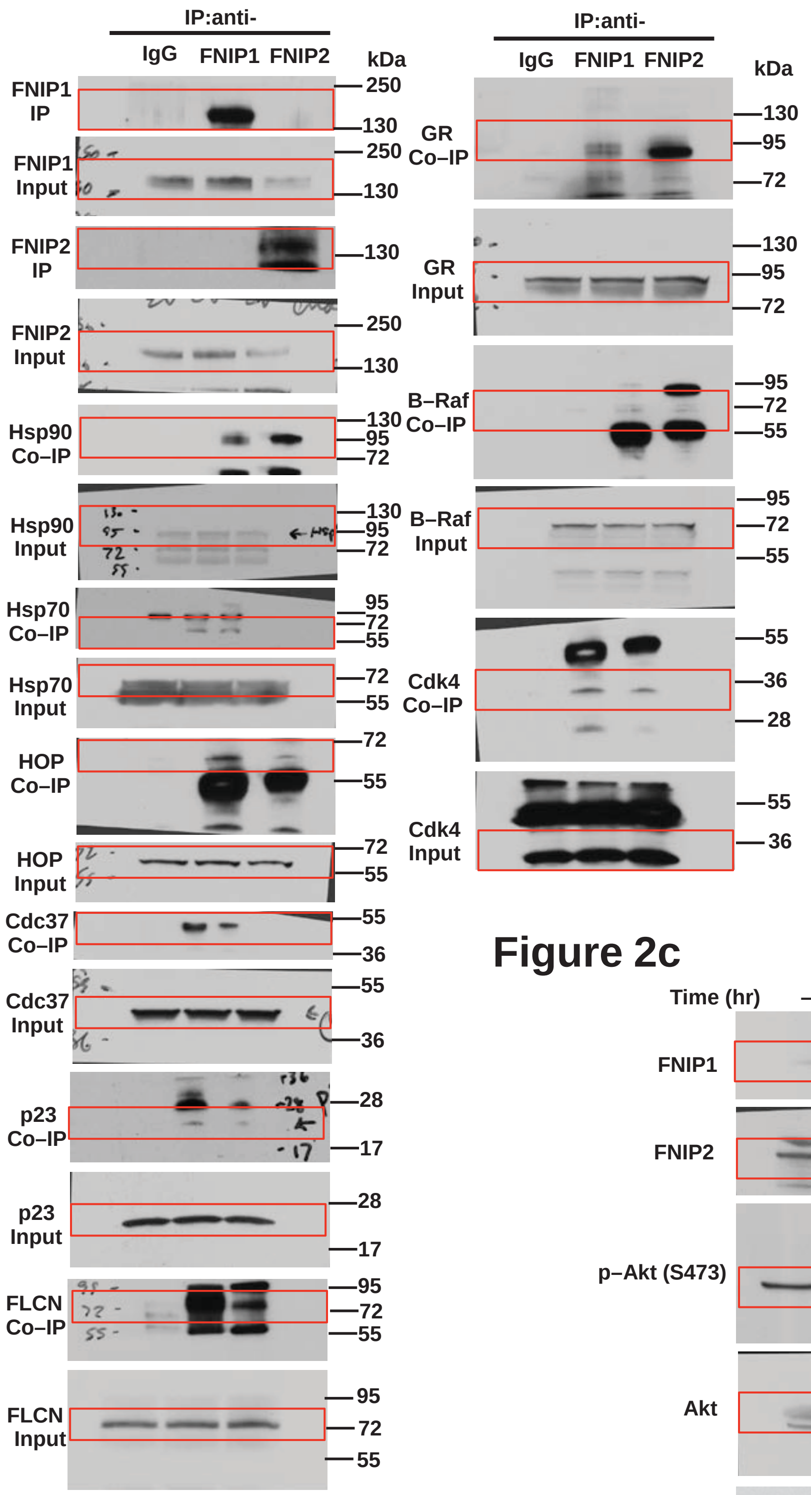

Figure 2b

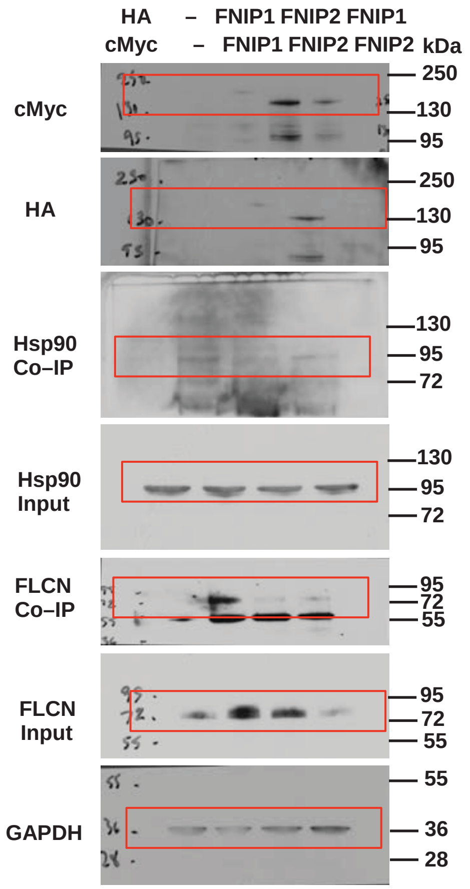

Figure 2c

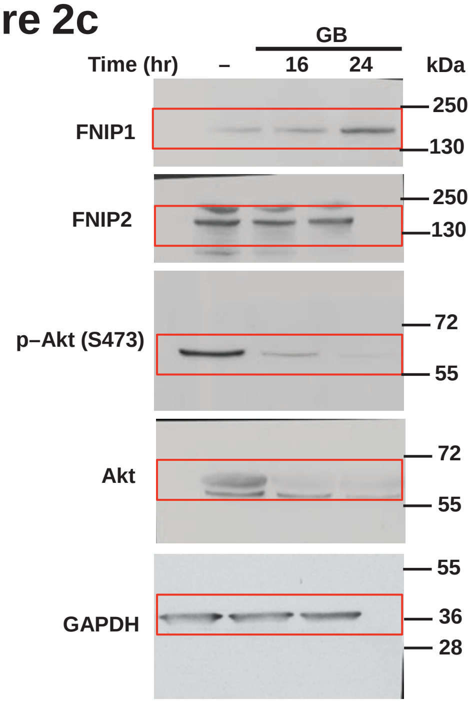

Figure 2d

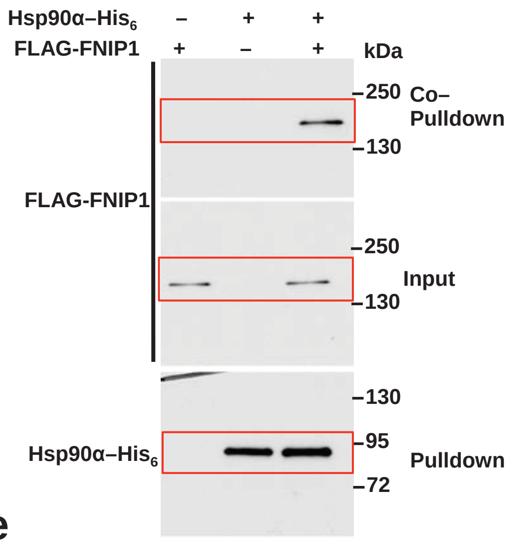

Figure 2e

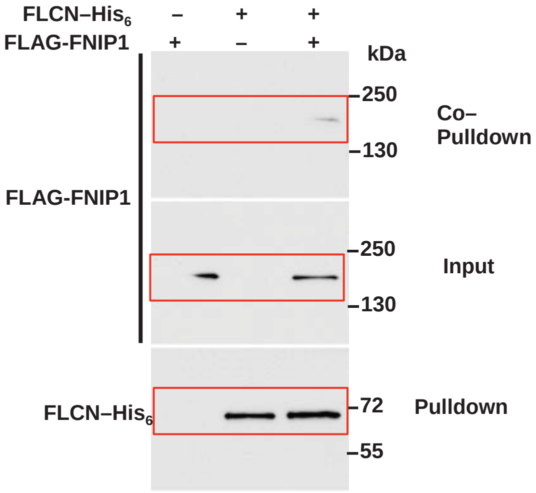

Figure 2f

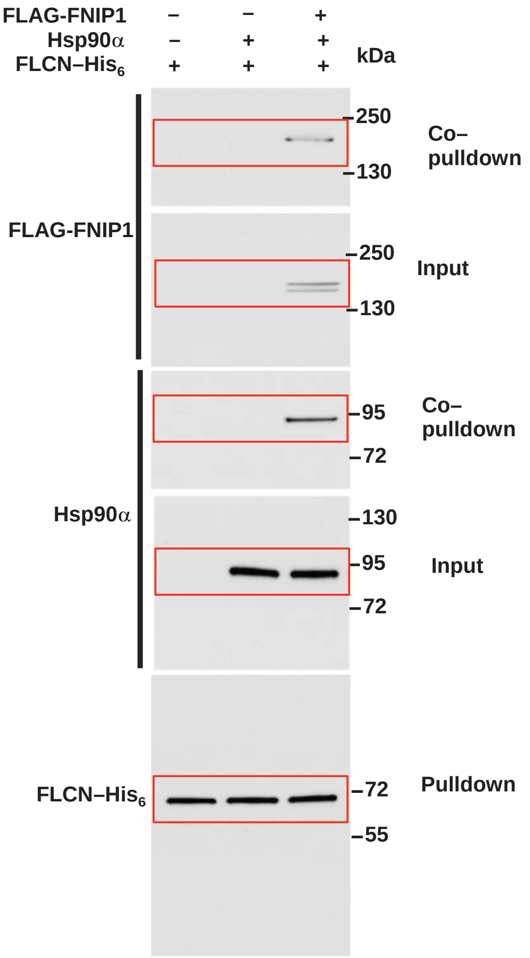

Figure 3a

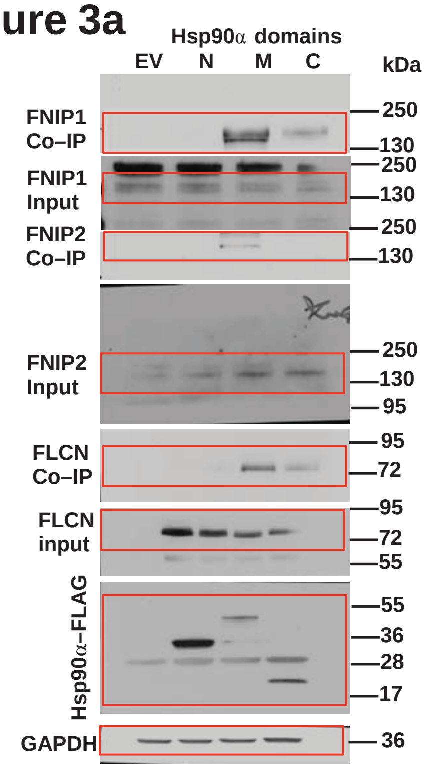

Figure 3b

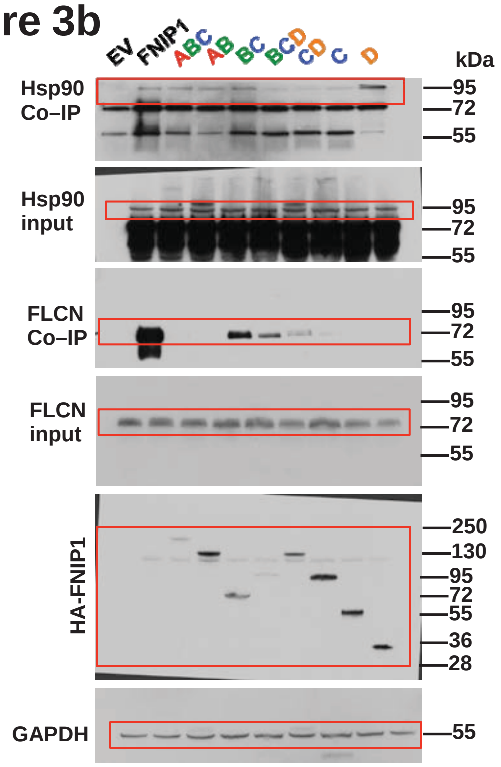

Figure 3c

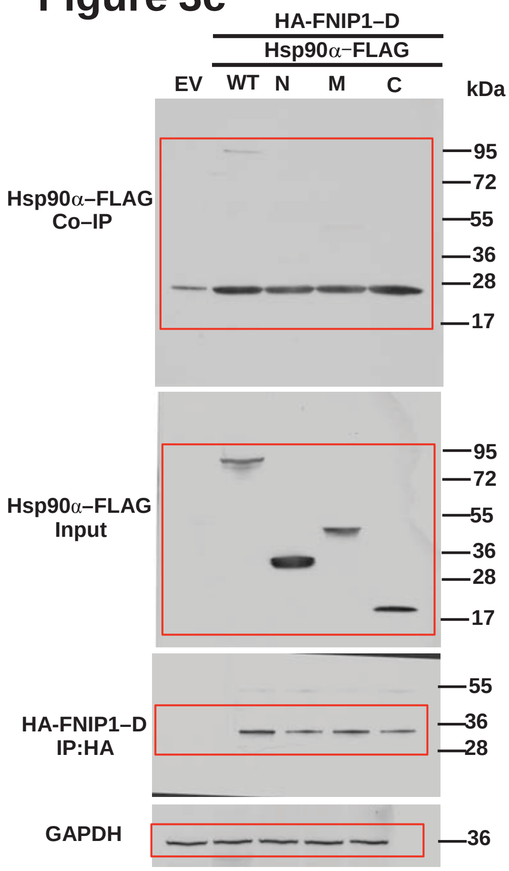

Figure 4a

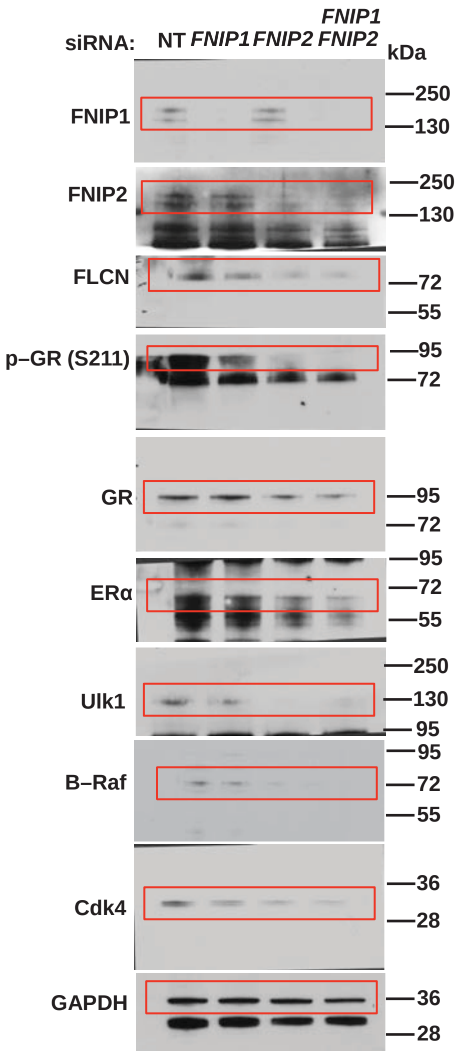

Figure 4b

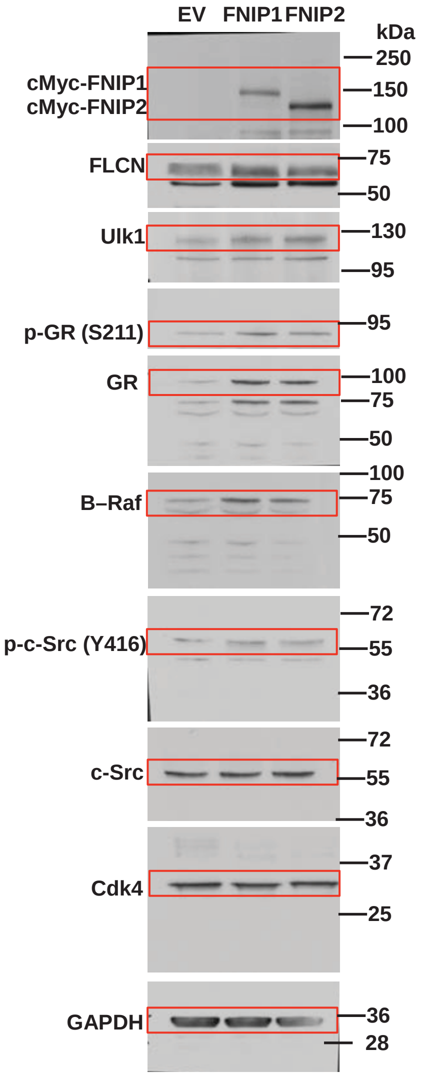

Figure 4c

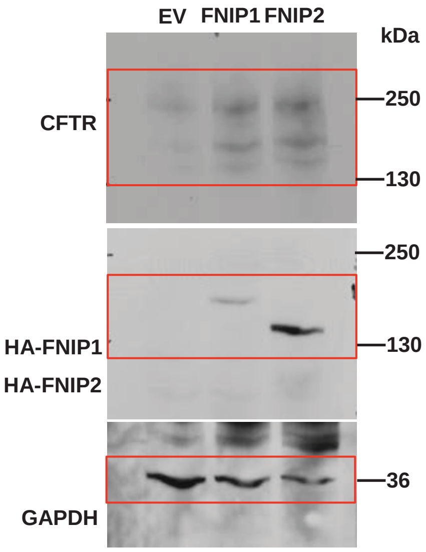

Figure 5a

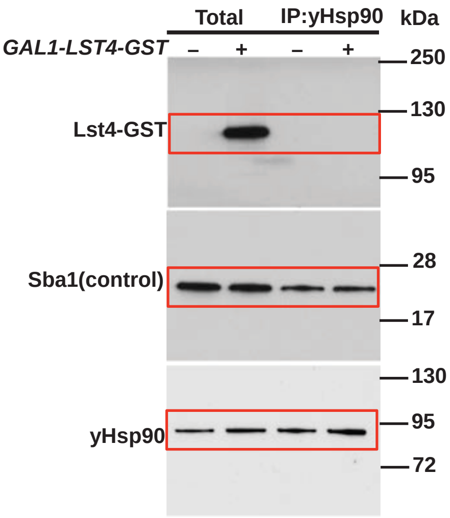

Figure 5d

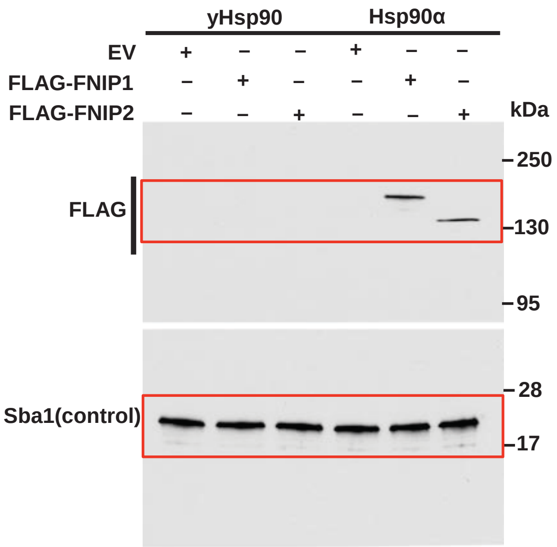

Figure 6a

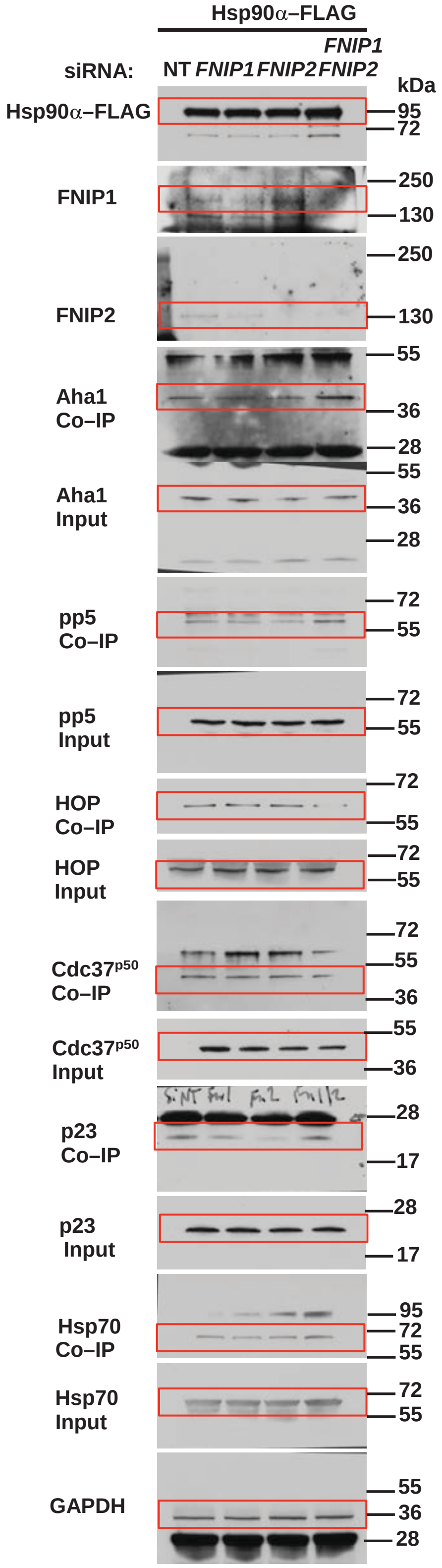

Figure 6b

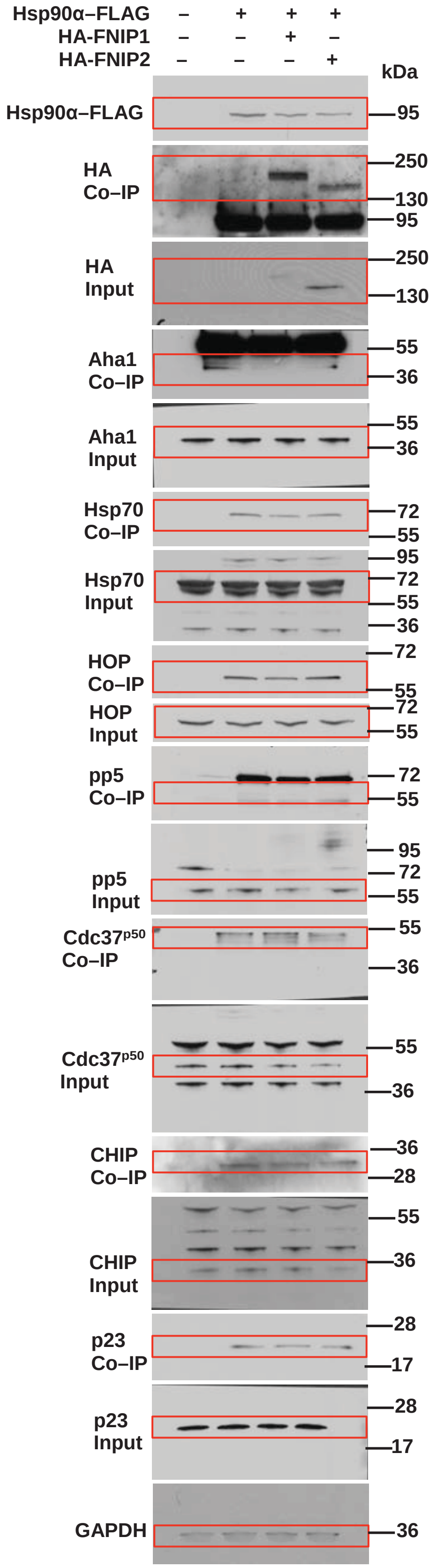

Figure 6d

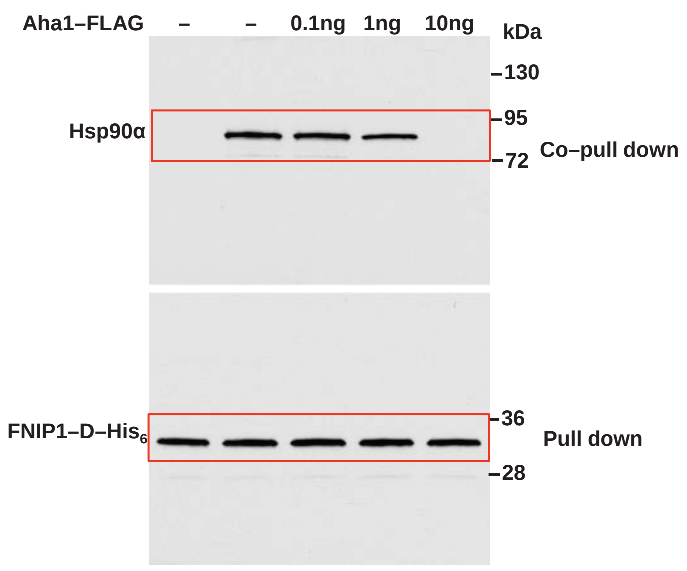

Figure 6e

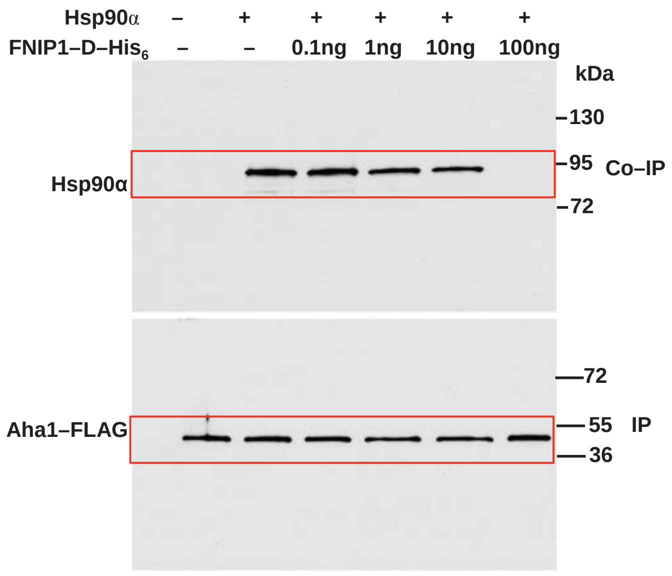

Figure 7b

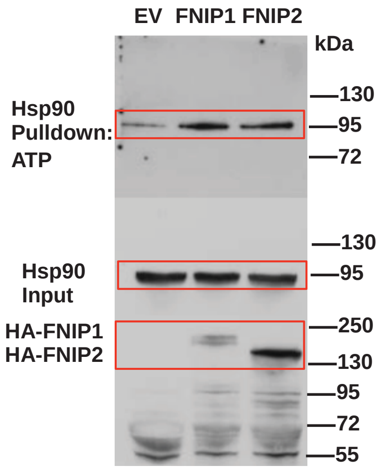

Figure 7d

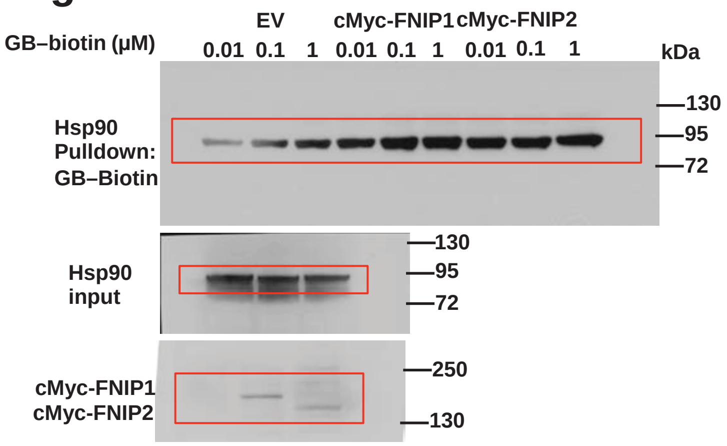

Figure 7f

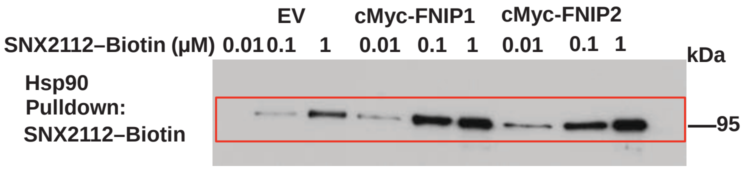

Figure 7g

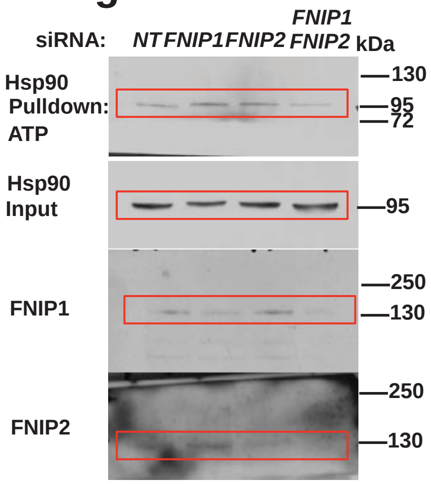

Figure 7h

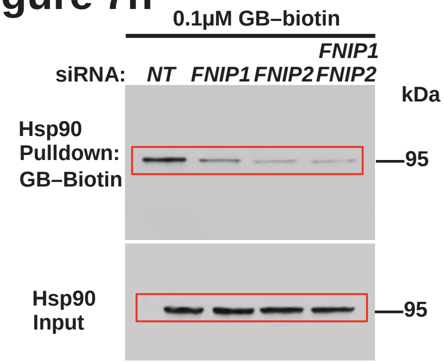

Figure 8a

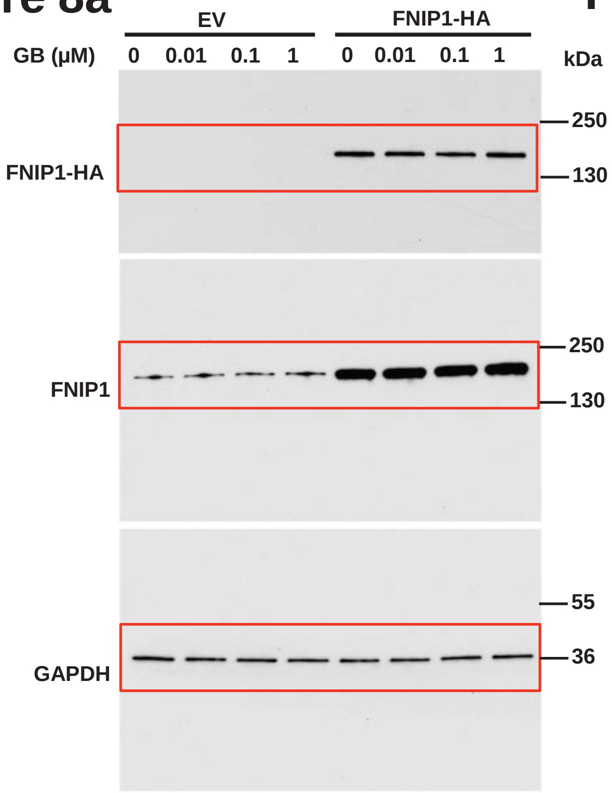

Figure 8b

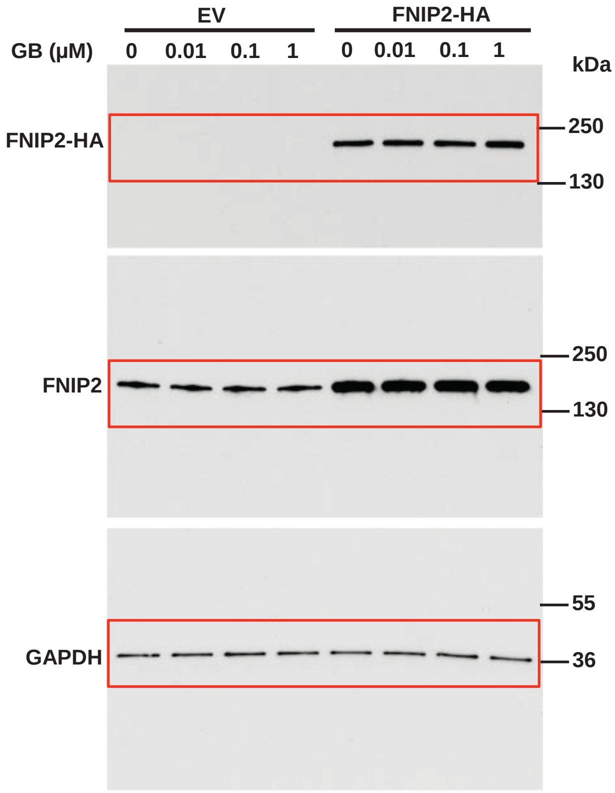

Figure 8c

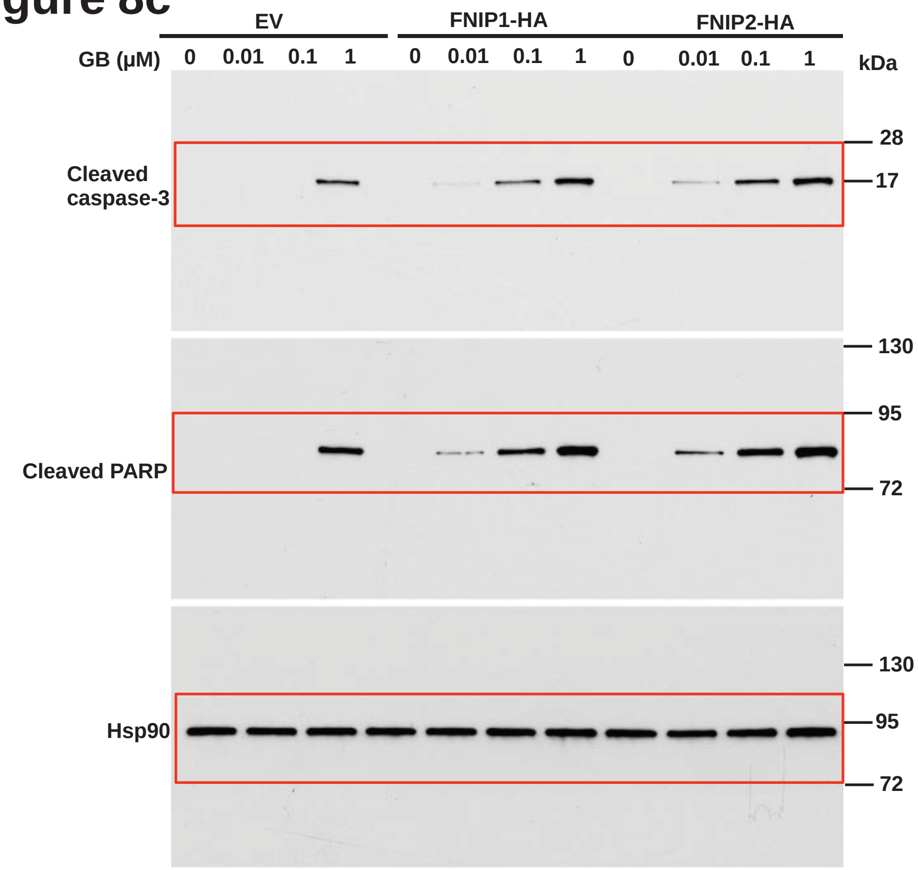

Figure 8d

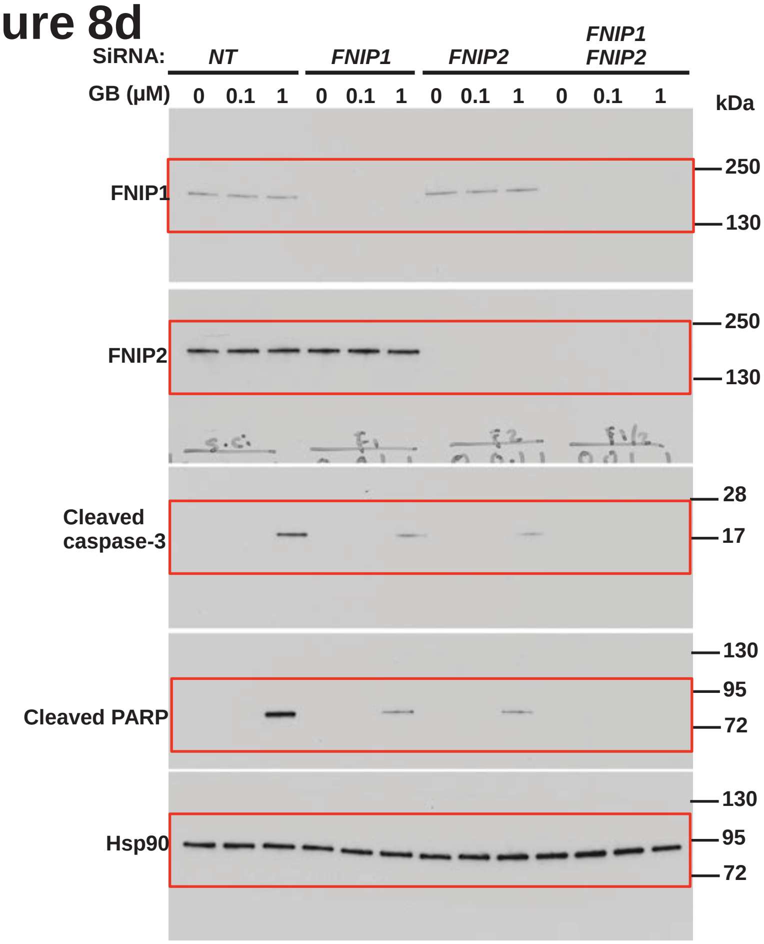

Figure 9a

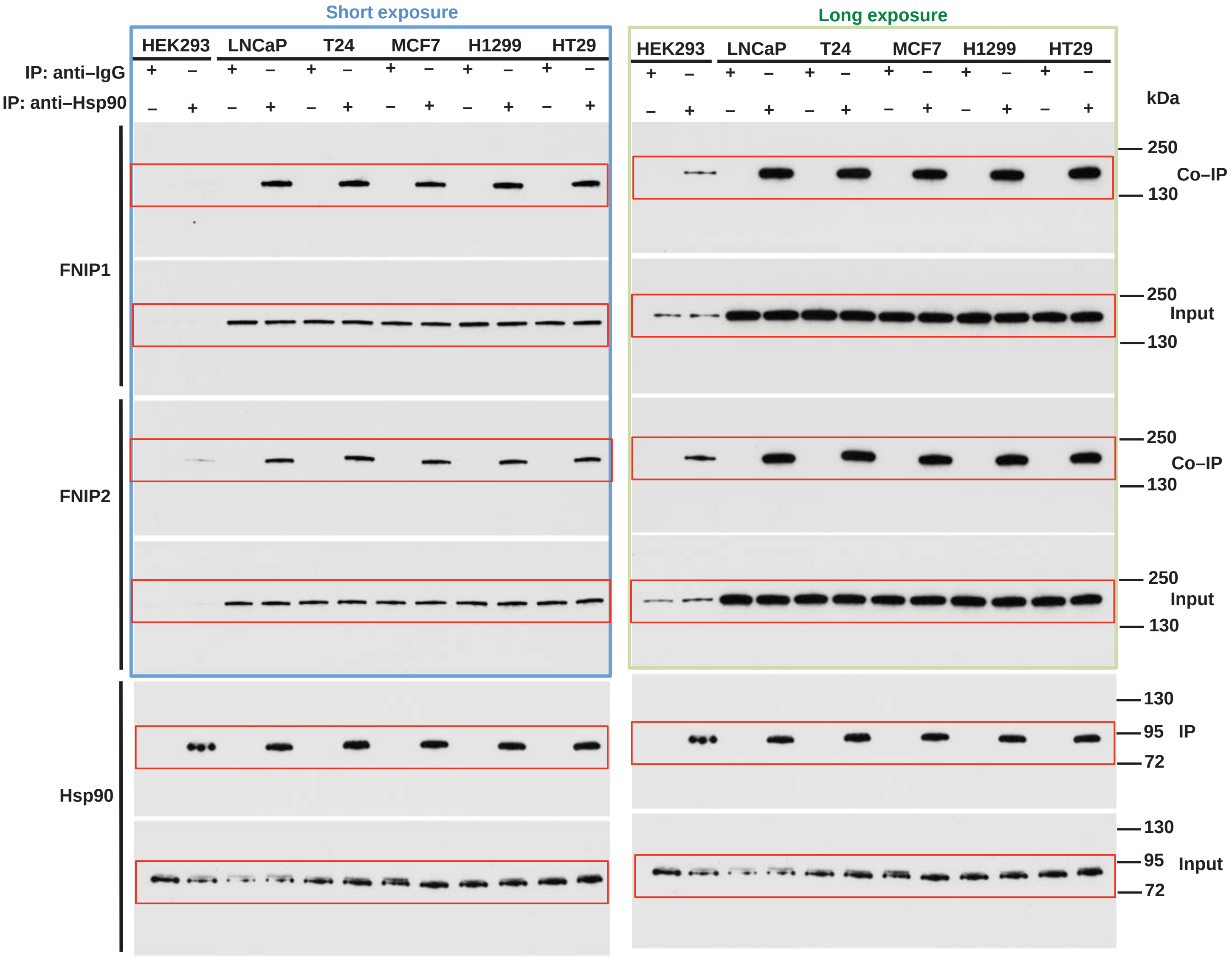

Short exposure
Long exposure
HEK293
LNCaP
T24
MCF7
H1299
HT29
IP: anti–IgG
IP: anti–Hsp90
kDa
FNIP1
FNIP2
Hsp90
250
130
Co–IP
Input
95
72
IP

Figure 9b Figure 9c Figure 9d Figure 9e

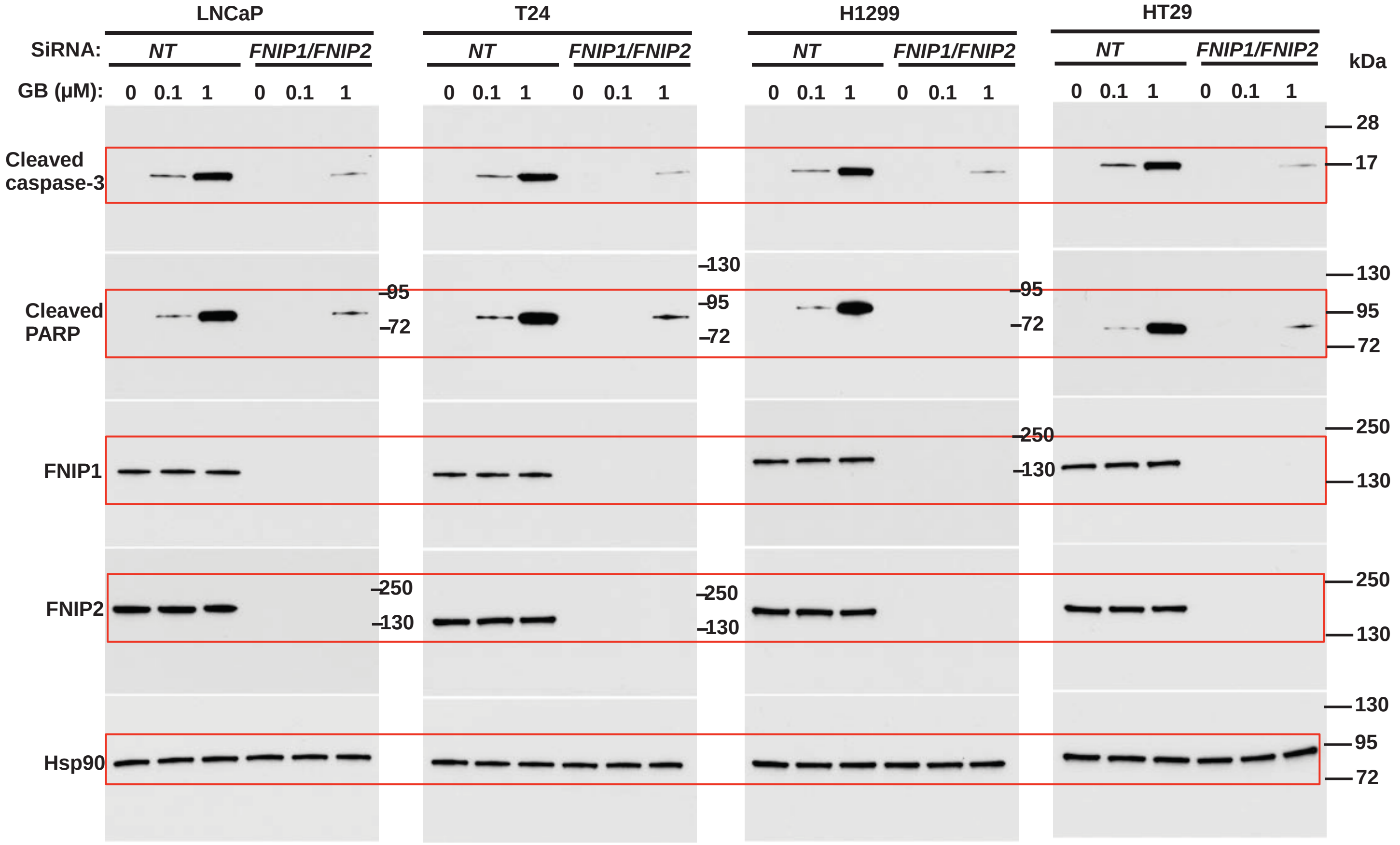

Figure 9f

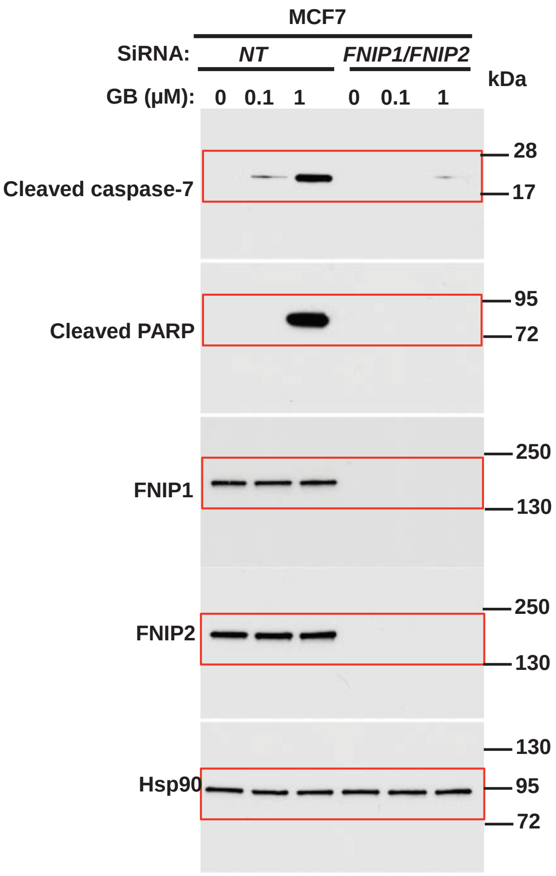

Figure 10a-d

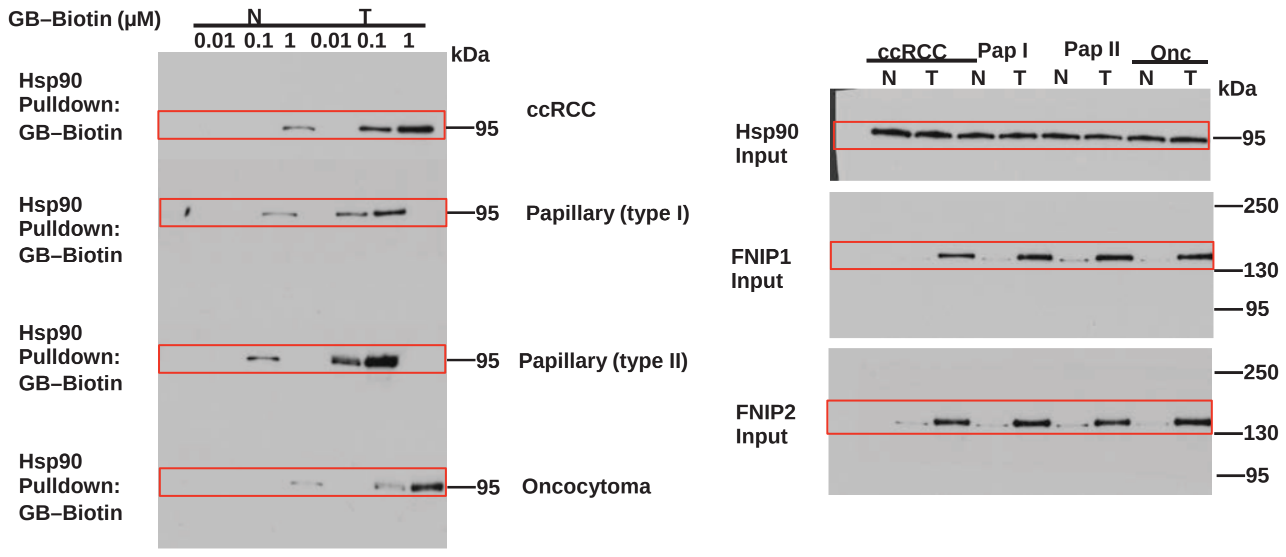

Figure 10e

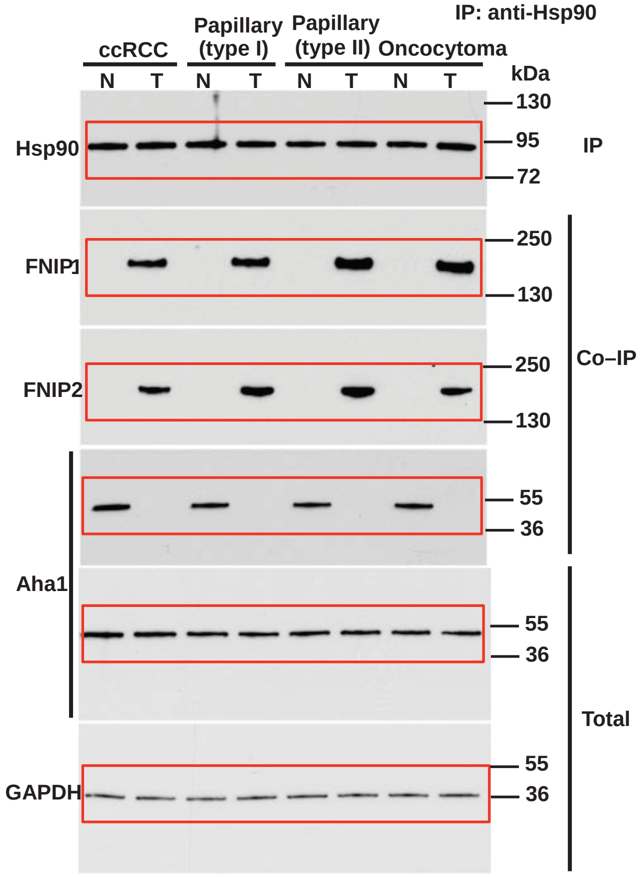

Figure 10f

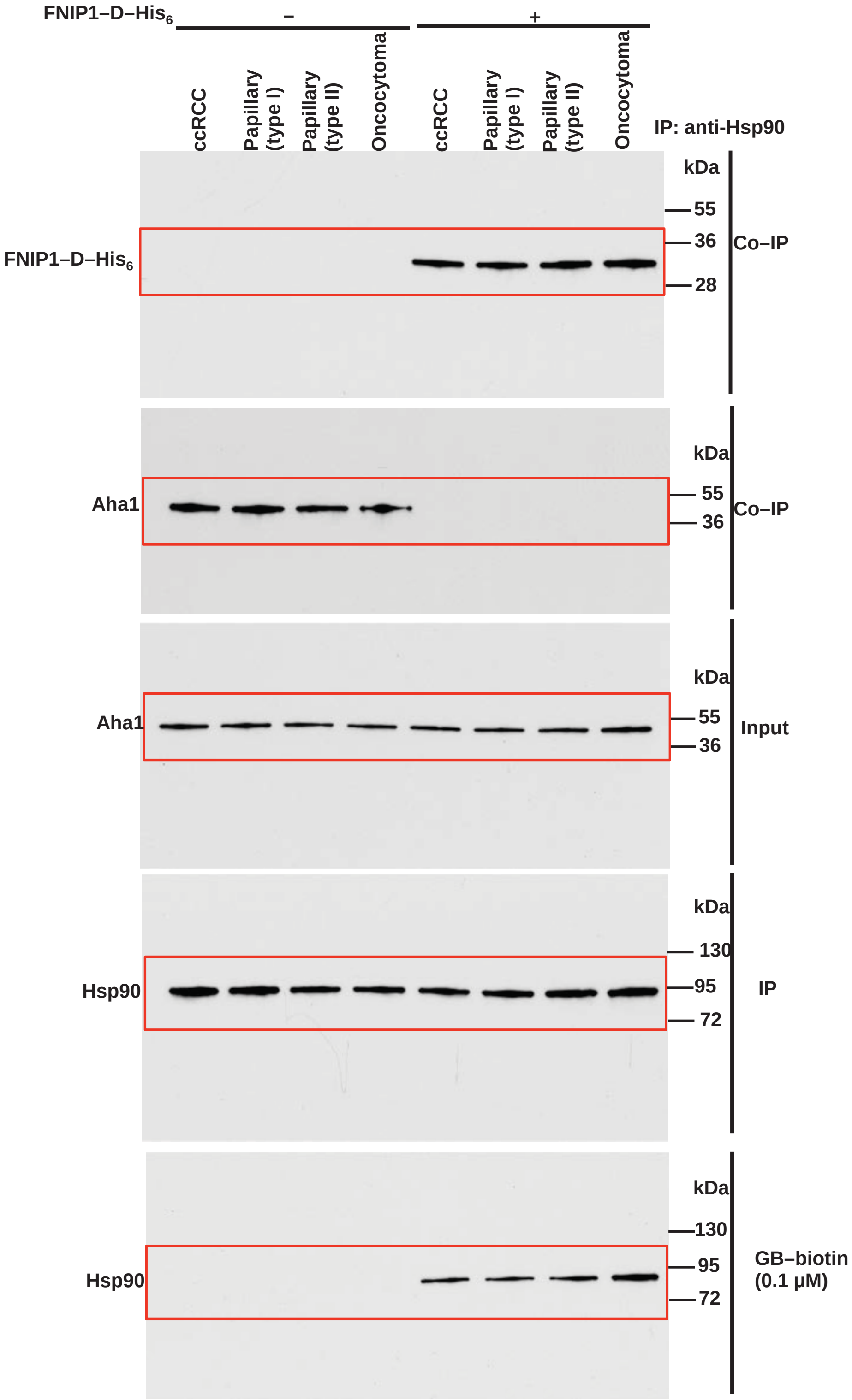
FNIP1–D–His6
–
+
ccRCC
Papillary (type I)
Papillary (type II)
Oncocytoma
ccRCC
Papillary (type I)
Papillary (type II)
Oncocytoma
IP: anti-Hsp90
kDa
55
36
28
Co–IP
FNIP1–D–His6
Aha1
kDa
55
36
Co–IP
Aha1
kDa
55
36
Input
Hsp90
kDa
130
95
72
IP
Hsp90
kDa
130
95
72
GB–biotin (0.1 μM)

Supp. Figure 1a

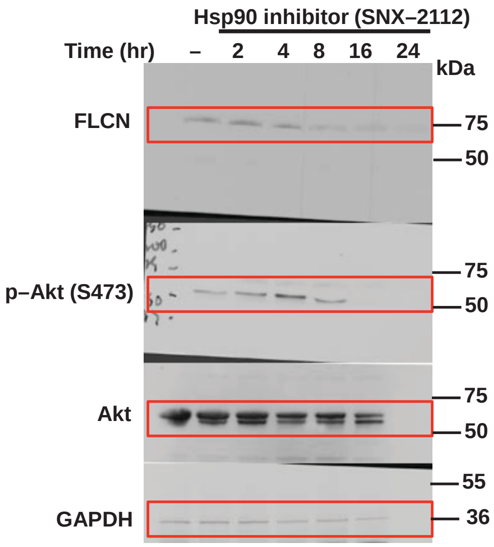

Supp. Figure 1b

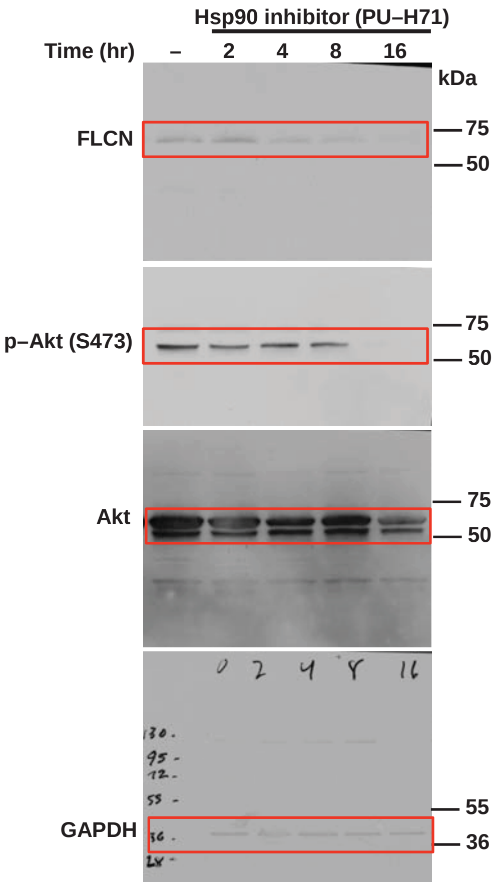

Supp. Figure 1a

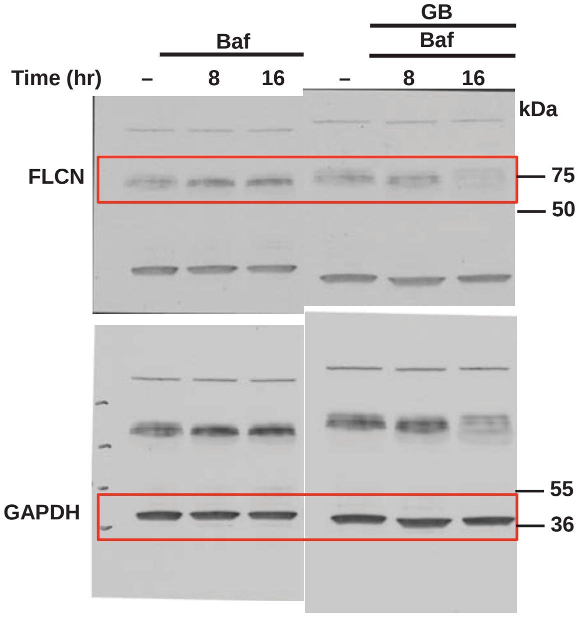

Supp. Figure 2a

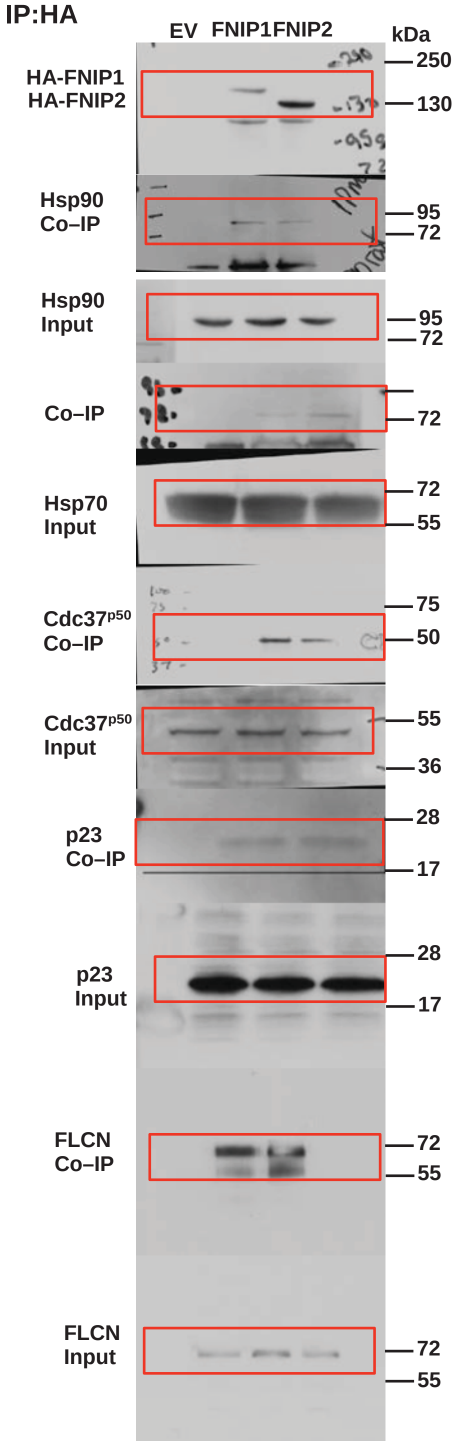

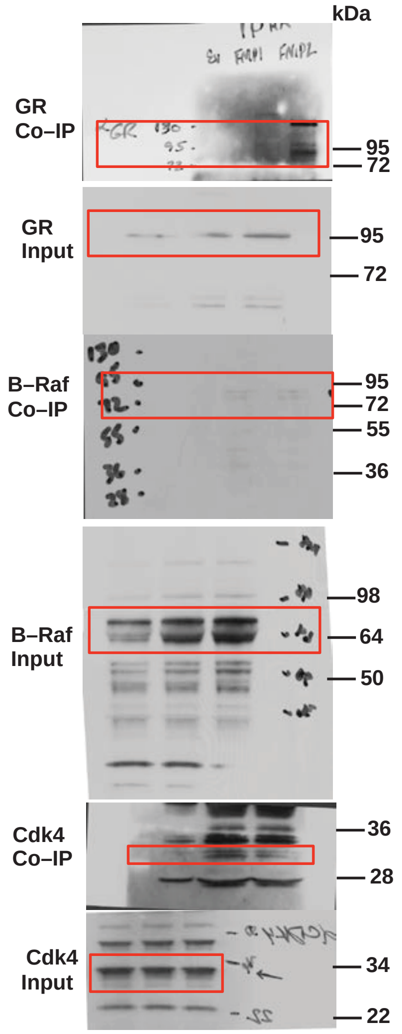

Supp. Figure 2c

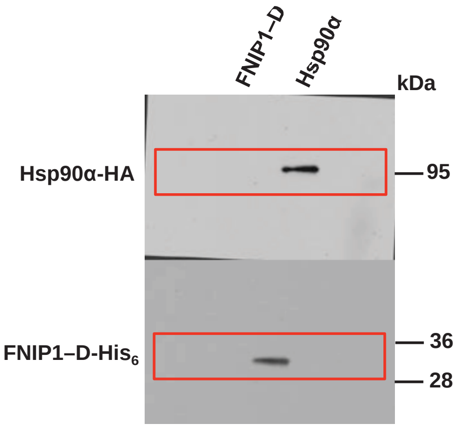

Supp. Figure 4a

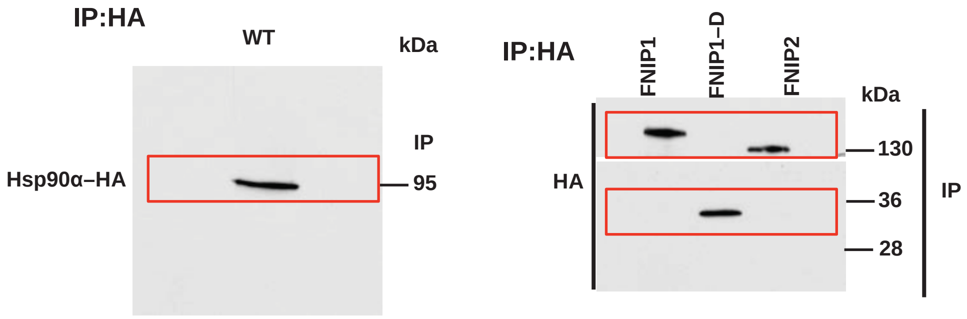

Supp. Figure 5a

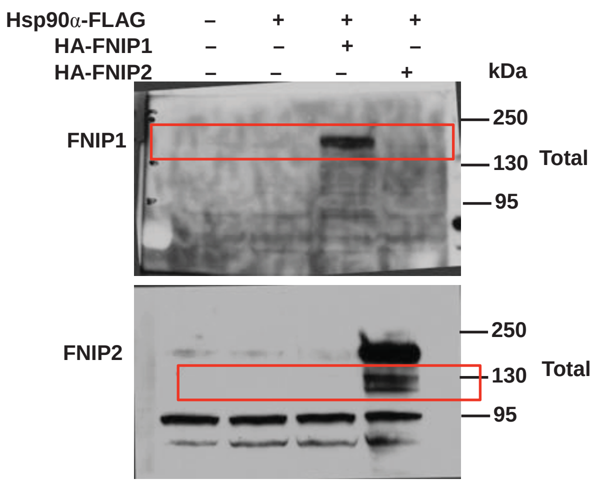

Supp. Figure 5f

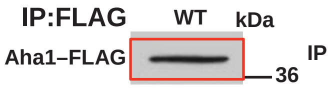

Supp. Figure 5h

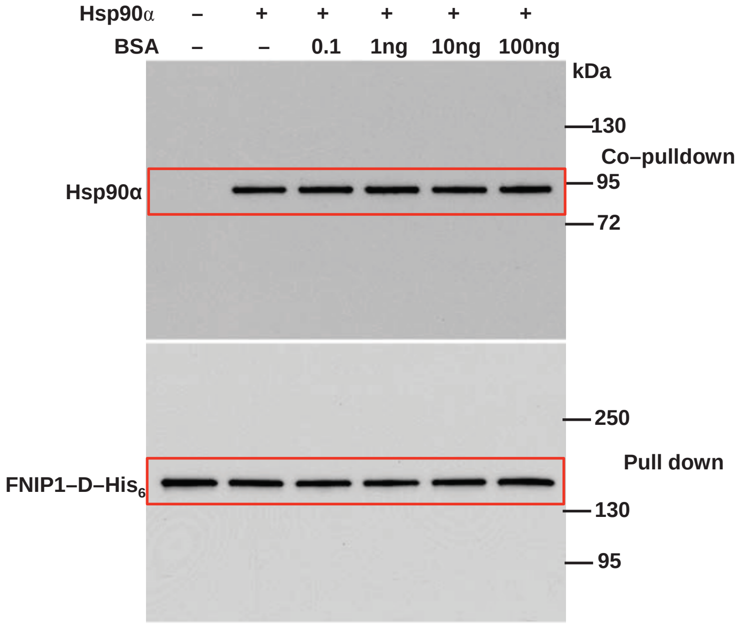

Supp. Figure 5i

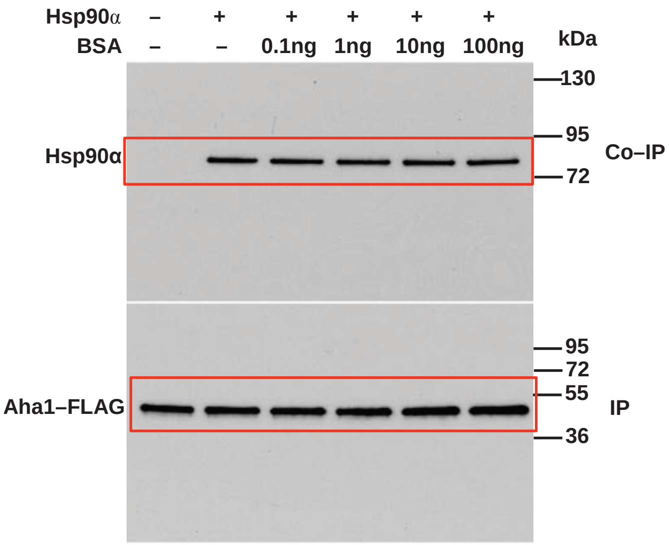

Supplementary Table 1. Cellular proteins associated with FLCN.

Protein	MW	Protein Score	C.I.%	Total Ion Score	C.I.%	Peptides	Accession Number
Spliceosomal protein SAP 155	145723	158	100	124	100	HFWQHR<br>VLPPPAGYVPIR<br>MTPPIKDLLPR<br>RATVNTFGYIAK<br>LDDLVRPYVHK<br>DTPGHGSGWAETPR<br>IVDDLKDEAEQYRK<br>AGELKVVNGAAASQPPSK<br>IWDPTPSHTPAGAATPGR<br>EWMRICFELLELLK<br>TPDPKMNVRTYMDVMR<br>DYIYAVTPLLEDALMDR<br>LLVDVDESTLSPEEQKER<br>NRPLSDEELDAMFPEGYK<br>EDEYKKHRRTMISPER<br>IYNSIYIGSQDALIAHYPR<br>AIGYLIPLMDAEYANYYTR<br>IYNDDKNTYIRYELDYIL<br>KLTATPTPLGGMTGFHMQTEDR<br>KPGYHAPWALLNDIPQSTEQYDPFAEHRPPK<br>EIQGKKAALDEAQGVGLDSTGYYDQEIYGGSDSR	gi|4033735
Splicing factor 3b, subunit 3, 130kDa	135432	N/A	N/A	24	88	FLAVGLVDNTVR	gi|46362557
Heat shock protein HSP 90-alpha	98180	89	100	83	100	VILHLKEDQTEYLEER<br>HSQFIGYPITLFVEKER	gi|153792590
Heat shock 70 kDa protein 1A/1B	70009	222	100	173	100	DEFEHK<br>LLQDFFNGR<br>DAGVIAGLNVLR<br>LVNHFVEEFKR<br>LLQDFFNGRDLNK<br>ATAGDTHLGGEDFDNR<br>IINEPTAAAIAYGLDR<br>NQVALNPQNTVFDAKR<br>LDKAQIHDLVLVGGSTR<br>RLIGRKFGDPVVQSDMK<br>GRLSKEEIERMVQEAEK<br>STGKANKITITNDKGRLSK<br>QTQIFTTYSDNQPGVLIQVYEGER<br>TLSSSTQASLEIDSLFEGIDFYTSITR<br>MKEIAEAYLGYPVTNAVITVPAYFNDSQR<br>VEIIANDQGNRTTPSYVAFTDTERLIGDAAK	gi|167466173
Chaperonin containing TCP1, subunit 8 (theta)	59440	171	100	156	100	GYEIACRK<br>FAEAFEAIPR HFSGLEEAVYR<br>LFVTNDAATILR<br>HEKEDGAISTIVLR<br>MALHVPKAPGFAQMLK<br>VDQIIMAKPAGGPKPPSGKK<br>YNIMLVRLNSKWDLRR<br>VADMALHYANKYNIMLVR<br>TAEELMNFSKGEENLMDAQVK<br>ELAQTTRTAYGPNGMNKMVINHLEK<br>RLVPGGGATEIELAKQITSYGETCPGLEQYAIKK	gi|119630329

T-complex protein 1 subunit beta	57424	94	100	27	92	VPDHHPC<br>LIEEVMIGEDK GATQQILDEAER<br>LLTHHKDHFTK<br>QDLMNIAGTTLSSK<br>TPGKEAVAMESYAKALK<br>RQVLLSAAEAAEVILR<br>EALLSSAVDHGSDEVKFR<br>IHPQTIIAGWREATKAAR<br>LALVTGGEIASTFDHPELVK<br>VDSTAKVAEIEHAEKEKMKEK<br>EGTIGDMAILGITESFQVKR<br>VQDDEVGDGTTSVTVLAAELLR<br>ALKMLPTIIADNAGYDSADLVAQLR<br>RIENAKILIANTGMDTDKIKIFGSR	gi|197692147
T-complex protein 1 subunit eta	59329	N/A	N/A	60	100	QVKPYVEEGLHPQIIIR	gi|5453607
T-complex protein 1 subunit delta	57888	N/A	N/A	47	100	ALIAGGGAPEIELALR	gi|38455427
Activator of 90 kDa heat shock protein ATPase homolog	38250	192	100	131	100	WGEGDPR<br>WIVEER<br>TQARPVGVK<br>GIPAPEEERTR<br>LDGEASINNRK<br>NGETELCMEGR<br>LIFFYEWSVK<br>YYFEGIKQTFGYGAR<br>VFTTQELVQAFTHAPATLEADRGGK<br>ADATNVNNWHWTERDASNWSTDKLK	gi|6912280
E3 ubiquitin-protein ligase CHIP	34834	119	100	107	100	LIAAERER<br>VGHFDPVTR<br>NPLVAVYYTNR<br>LNFGDDIPSALR<br>RIHQESELHSYLSR<br>ISFELMREPCITPSGITYDR	gi|56181387
Ribosomal protein L10	25067	68	96	35	99	GAFGKPQGTVAR<br>VRLHPFHVIR ICANKYMVKSCGK<br>INKMLSCAGADRLQTGMR<br>SRFCRGVPDAKIRIFDLGR<br>LQTGMRGAFGKPQGTVARVHIGQVIMSIRTK	gi|119593144

Supplementary Table 2. siRNA sequences.

Name	Sequences
FNIP1-1	CCCAGGAAUUGUGCGGAAA
FNIP1-2	AAGCAUAACUUGUCUCCAA
FNIP1-3	GCAAAGACUCAUCCAUAUA
FNIP1-4	GCACAAAUACUCCUUUAAU
FNIP2-1	GGACAGUGGCAUUGCUCGA
FNIP2-2	AUAAAGAGGCACCGCAAGA
FNIP2-3	GAAAGAAUUAGGUGUCGUA
FNIP2-4	CCUCGUGGCUGGUGCGAAU
NON-TARGETING POOL-1	UGGUUUACAUGUCGACUAA
NON-TARGETING POOL-2	UGGUUUACAUGUUGUGUGA
NON-TARGETING POOL-3	UGGUUUACAUGUUUUCUGA
NON-TARGETING POOL-4	UGGUUUACAUGUUUUCCUA

Supplementary Table 3. Primer sequences. Restriction sites are highlighted in green. Epitope sequences are highlighted in blue.

Primer	Sequence
FLAG–Hsp90–N–BamHI–F	TATGCGGGATCCATGGATTACAAGGATGACGACGATAAGGGACCTGAGGAAACCCAGACCCAAGAC
FLAG–Hsp90–N–XhoI–R	CTCGGTCTCGAGTTACACAAAAAGAGTAATGGGATATCCAATAAACTGAGA
FLAG–Hsp90–M–BamHI–F	TATGCGGGATCCATGGATTACAAGGATGACGACGATAAGGGAGATCAAGAAGAGCTCAACAAAACA
FLAG–Hsp90–M–XhoI–R	CTCGGTCTCGAGTTATCTTAGGGCTTGAGCTTTCATGATTCTCTCCATGTTT
FLAG–Hsp90–C–BamHI–F	TATGCGGGATCCATGGATTACAAGGATGACGACGATAAGGGAGACAACTCAACAATGGGTTACATG
FLAG–Hsp90–C–XhoI–R	CTCGGTCTCGAGTTAGTCTACTTCTTCCATGCGTGATGTGTCGTCATCTCC
FLCN-Flag-Spe1	CTCGTACACTAGTATGGACTACAAGGACGACGATGACAAGAATGCCATCGTGGCTCTCTGCC
FLCN-R-Xho1	CTAAGGACTCGAGTCAGTTCCGAGACTCCGAGGCTGTG
Fnip1–NheI–F	TATGCGCTAGCGCCCCTACGCTGTTCCAGAAGCTCT
Fnip1–XhoI–R	CTCGGTCTCGAGTTAAAGGAGTATTTGTGCAACATATGGAG
Fnip2–NheI–F	TATGCGCTAGCGCCCCGACCCTGCTCCAGAAGCTCTTC
Fnip2–XhoI–R	CTCGGTCTCGAGTTATAAGAGTATTTGAGCCACATAAGGA
Fnip1-NotI-F	TATGCGCGGCCGCGCCCCTACGCTGTTCCAGAAGCTCT
Fnip2-NotI-F	TATGCGCGGCCGCGCCCCGACCCTGCTCCAGAAGCTCTTC
Fnip1–KpnI–FLAG–F	TATGCGGTACCATGGATTACAAGGATGACGATGACAAGGGATCCGCCCCTACGCTGTTCCAGAAGCTCT
Fnip2–KpnI–FLAG–F	TATGCGGTACCATGGATTACAAGGATGACGATGACAAGGGATCCGCCCCGACCCTGCTCCAGAAGCTCT
Fnip1–SpeI–cMyc–F	TATGCACTAGTATGGAACAAAAGTTAATCTCCGAAGAAGATTTAGCCCCTACGCTGTTCCAGAAGCTCTT
Fnip2–SpeI–cMyc–F	TATGCACTAGTATGGAACAAAAGTTAATCTCCGAAGAAGATTTAGCCCCGACCCTGCTCCAGAAGCTCTTCAA
